# Cyclodextrin-Based Contrast Agents for Medical Imaging

**DOI:** 10.3390/molecules25235576

**Published:** 2020-11-27

**Authors:** Yurii Shepelytskyi, Camryn J. Newman, Vira Grynko, Lauren E. Seveney, Brenton DeBoef, Francis T. Hane, Mitchell S. Albert

**Affiliations:** 1 Chemistry and Materials Science Program, Lakehead University, Thunder Bay, ON P7B 5E1, Canada; yshepely@lakeheadu.ca (Y.S.); vgrynko@lakeheadu.ca (V.G.); 2 Thunder Bay Regional Health Research Institute, Thunder Bay, ON P7B 6V4, Canada; francishane@gmail.com; 3 Biology Department, Lakehead University, Thunder Bay, ON P7B 5E1, Canada; cjnewma1@lakeheadu.ca; 4 Chemistry Department, University of Rhode Island, Kingston, RI 02881, USA; laurenseveney@uri.edu (L.E.S.); bdeboef@uri.edu (B.D.); 5 Chemistry Department, Lakehead University, Thunder Bay, ON P7B 5E1, Canada; 6 Northern Ontario School of Medicine, Thunder Bay, ON P7B 5E1, Canada

**Keywords:** medical imaging, contrast agents, α-cyclodextrin, β-cyclodextrin, γ-cyclodextrin, MRI, PET, CT, SPECT, PAI

## Abstract

Cyclodextrins (CDs) are naturally occurring cyclic oligosaccharides consisting of multiple glucose subunits. CDs are widely used in host–guest chemistry and biochemistry due to their structural advantages, biocompatibility, and ability to form inclusion complexes. Recently, CDs have become of high interest in the field of medical imaging as a potential scaffold for the development of a large variety of the contrast agents suitable for magnetic resonance imaging, ultrasound imaging, photoacoustic imaging, positron emission tomography, single photon emission computed tomography, and computed tomography. The aim of this review is to summarize and highlight the achievements in the field of cyclodextrin-based contrast agents for medical imaging.

## 1. Introduction

**Figure 1:**
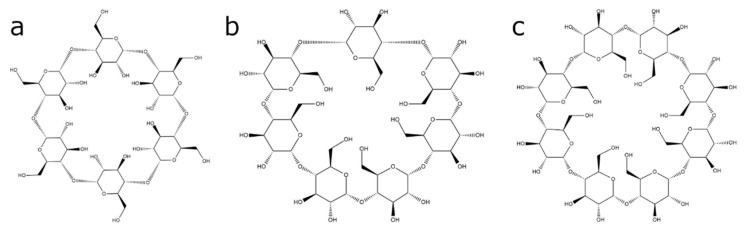
Chemical structure of (**a**) α-cyclodextrin, (**b**) β-cyclodextrin, and (**c**) γ-cyclodextrin.

Cyclodextrins (CDs) are chemically stable naturally occurring cyclic oligosaccharides consisting of multiple glucose subunits connected by α-1,4 glycosidic bonds [1,2]. There are three main types of CDs that contain six (α-CD), seven (β-CD), and eight (γ-CD) glucose subunits in a ring (Figure 1). These cyclodextrin macromolecules are cone-shaped with a hydrophobic interior cavity and polar exterior surface [3]. Due to their non-toxic nature [1,4,5,6] and water solubility [7,8,9], CDs became widely used in various biomedical fields such as drug solubilization [7,10,11,12], drug delivery [8,13,14,15,16,17], and nucleic acid transfer [18,19,20].

Compared to other macrocyclic hosts, cyclodextrins are by far the most extensively used in host–guest chemistry applications and medical imaging [21,22,23,24,25]. They tend to be the macrocycle of choice due to their structural advantages and robust ability to form inclusion complexes [21,26]. An inclusion complex is formed when a guest molecule, commonly a small drug, is partially or fully encapsulated inside the host’s interior cavity [1,3]. In the case of cyclodextrins, their preferred guest molecules tend to be hydrophobic, making them suitable for binding in the hydrophobic interior. Therefore, cyclodextrins possess the ability to form inclusion complexes with a wide variety of hydrophobic guest molecules [26,27,28]. Formation of inclusion complexes, or molecular encapsulation, can affect the physiochemical properties of the drug or molecule itself, such as solubility and rate of dissolution [3]. CDs are often exploited because of this property in addition to enhancing water solubility of water-insoluble molecules [3]. The exterior of cyclodextrin is predominantly hydrophilic due to the extensive hydrogen bonding network, making it a biocompatible agent for a wide range of applications [1,2,3,6,7,8,9,10,11,12,13,14,15,16,17]. These structural factors are largely why CDs are favored when synthesizing inclusion complexes. Electronics and thermodynamics both play a role in determining if a CD will form an inclusion complex with a guest molecule [1]. The driving force for inclusion complexation involves various noncovalent interactions such as desolvation, or removal, of water molecules from the interior cavity and formation of Van der Waals, hydrophobic, and hydrogen bonding interactions [29]. The major driving forces for cyclodextrin complexation are van der Waals interaction and hydrophobic interaction, whereas hydrogen bonding and electrostatic interaction mostly affect the conformation of a particular inclusion complex [29]. CD’s ability to form inclusion complexes with small organic molecules has pioneered the field of supramolecular chemistry. More recently, CDs have successfully been used to build various molecular architectures such as catenanes, rotaxanes, pseudorotaxanes, polyrotaxanes, and other molecular machines [30,31].

During the past few decades, cyclodextrins have become of interest as contrast agents and potential biosensors for different medical imaging modalities [32]. In the context of imaging, CDs have been used primarily as a scaffold to support and/or solubilize smaller molecules that produce or quench a signal for enhanced imaging. However, in a few cases, the unique supramolecular nature of the CD is essential in producing the signal for imaging. Examples of CD-containing constructs that can be imaged by a wide variety of modern imaging technologies are discussed herein.

Magnetic resonance imaging (MRI) was the first imaging modality to utilize cyclodextrins as contrast agents [33]. CD-based MRI contrast agents produced contrast through the reduction in spin-lattice relaxation (T_1_) time of the water protons. There are two different established methods of synthesis of CD-based MRI contrast agents: (1) host–guest interactions between CD cavity and metal–organic complexes [33,34] and (2) by direct conjugation of CDs to the metal–organic complexes through the external hydroxyl groups of CD molecule [35,36]. The reduction in T_1_ relaxation for CD-based contrast agents, and therefore their contrast, is substantially stronger compared to the metal–organic complexes on their own [34,37].

Secondly, positron emission computed tomography (PET) utilizes CD-based molecular imaging probes [25]. The PET probes emit positron, which annihilates with a stationary electron from the surroundings producing two gamma-photons, which are detected [38]. The PET tracers based on CD can be divided in two classes. The first class contains CD-based nanoparticles (NPs) radiolabeled with either ^64^Cu [25] or ^18^F [39]. Another recently developed type of CD-based PET imaging agents contains CD molecules conjugated to the p-NCS-benzyl-NODA-GA (NODAGA) chelator labeled with ^68^Ga [40,41]. Followed by PET, the CD-based contrast agents were developed for single photon emission computed tomography (SPECT). SPECT contrast agents were created by radiolabeling of CD-based NPs either with ^99m^Tc [42] or ^125^I [43].

Recently, CDs were applied as contrast agents for ultrasound (US) and photo-acoustic imaging (PAI). The mechanism of contrast creation for US imaging relies on the substantial differences in the acoustic impedances between the biological tissue and the CD-based agents [44]. The mechanism of PA imaging is more complicated. The PA tracer absorbs the light with subsequent heating. Due to the temperature increase, the contrast agent undergoes thermoelastic expansion resulting in emission of the ultrasonic acoustic waves that can be detected by US receiver [45,46]. The developed CD photoacoustic contrast agents absorbed the light in the infrared range [47,48,49].

Lastly, multiple studies were conducted to evaluate the performance of the CD-based contrast agents for computed tomography (CT) [50,51,52]. All of the developments were focused on CD-based NPs that contained metal atoms (Au, Yb, Dy) [50,51,52,53]. The presence of the element with high atomic number rises up the X-ray absorption coefficient yielding to the contrast increase. These CD-based contrast agents demonstrated better performance compared to conventional iodine-based CT agents [50,51].

The purpose of this review is to provide an update on recent developments in CD-based contrast agents. A comparison of the developed contrast agents to the clinically available are presented as well as a comparison between different CD-based agents.

## 2. CD-Based Contrast Agents for MRI

MRI was the first medical imaging modality that began to develop CD-based contrast agents. The vast majority of the developed CD-based contrast agents reduce T_1_ relaxation of the surrounding protons and were used to produce substantial contrast on T_1_-weighted images. Nevertheless, several studies demonstrated the potential of T_2_ CD-based contrast agents as well. An overall summary of the achievements and developments in CD-based MRI contrast agents and molecular imaging probes is presented in detail below.

Due to the wide disfavor of traditional MRI probe modalities, mainly consisting of diethylenetriamine pentaacetic acid (DTPA) and dodecane tetraacetic acid (DOTA)-derived small molecule Gd(III) complexes, the continuous development and synthesis of innovative contrast agents is needed [54]. Cyclodextrin-based MRI contrast agents have gained notoriety in the last two decades and prove to be viable, robust candidates as potential MRI sensors for biomedical imaging [54]. In addition to their high biocompatibility and ability to form inclusion complexes, their large molecular weights, ease of functionalization and conjugation, and multivalent loading capacity make them well-suited as a new class of paramagnetic macromolecules [35,55,56]. CDs have proved to enhance contrast, sensitivity, and diagnostic imaging time [35]. This is attributed mainly due to their large molecular weights, which allow for longer and tunable rotational times [35,54].

### 2.1. Contrast Agents Based on Host–Guest Complexation between CDs and Metal–Organic Chelates

#### 2.1.1. In Vitro Studies of the Host–Guest CD-Based MRI Contrast Agents

The genesis of CD-based MRI contrast agents occurred in 1991 when Aime et al. [33] applied Freed’s theory [57] to predict that the relaxation rates of the solvent protons increase when the paramagnetic ion or complex is bound to a macrocycle. This principle became a fundamental base for the future development of CD-based MRI contrast agents. Aime et al. reported numerous results for the inclusion complexes formation between CD and gadolinium chelates such as DOTA and DTPA. Host–guest interaction is dependent on internal cavity size: α-CD did not demonstrate host–guest interaction, while β-CD non-covalently bonded with DOTA/DTPA with significant increase in proton relaxivity in vitro (reciprocal spin-lattice relaxation time (1/T_1_)) observed for both β-CD-DOTA and β-CD-DTPA contrast agents [33]. Subsequent studies utilizing Gd(III)-bis(benzylamide)diethylenetriaminepentaacetic acid (BBA-DTPA) [34], gadolinium (III) 3,6,9,15-tetraazabi-cyclo[1,3,9]pentadeca-1(15),11,13-triene-3,6,9-triacetic acid (PCTA) complexed with poly-β-CD [58], and DOTA-benzyloxymethyl (DOTA(bom)_3_) [59] demonstrated increasing water proton relaxivity due to the larger quantity of paramagnetic complexes accumulating in the region of interest due to interactions with a β-CD polymers [59]. The relaxivity of the developed poly- β-CD contrast agents exceeded up to six times the clinical analogues (r_1_ = 61 mM^−1^s^−1^) [59]. Furthermore, the results of modified Gd(III)-PCTA in blood serum suggested the possibility of application of this developed CD-based contrast agent in MRI angiography [58]. Despite such promising results, the relaxation measurements were conducted at 20 MHz NMR spectrometer at 0.5 T magnetic field far below the clinical MRI magnetic fields (1.5 or 3.0 T).

Larger CD-based NPs have also been the subject of recent research. These NPs have the potential for lower toxicity [60] compared to Gd-CD-based contrast agents. Examples of a proposed β-CD-based Gd-loaded NPs include a supramolecular assembly between DO3A-based gadolinium chelate (conjugated to adamantane through an acetamide spacer), poly-β-CD, and modified dextran [60] and cleavable β-CD-based Gd(III)-loaded nanocapsules, a promising agent as a redox-sensitive MRI contrast agent [61]. Besides toxicity reduction in Gd chelates, the proposed NPs are utilized the concept of further increasing of the number of Gd(III) atoms per unit of contrast agent resulting in increasing relaxivities.

#### 2.1.2. In Vivo Imaging of the Host–Guest CD-Based Contrast Agents

The performance of some CD-based MRI imaging agents has been evaluated in vivo. Lahrech et al. demonstrated imaging of C6 glioma rats using a Gd-α-CD complex to quantify cerebral blood volume [62]. Although performance of Gd-α-CD contrast agents was significantly better compared to Gd-DOTA in terms of relaxation rates (r_1_ = 7.3 mM^−1^s^−1^ at 9.4 T), the developed supramolecular agent did not accumulate in tumors and almost no enhancement was observed on T_1_-weighted images during the first hour after Gd-α-CD injection. On the contrary, the CBV fraction was successfully measured using rapid steady state T_1_ method and Gd-α-CD contrast agent.

**Figure 2:**
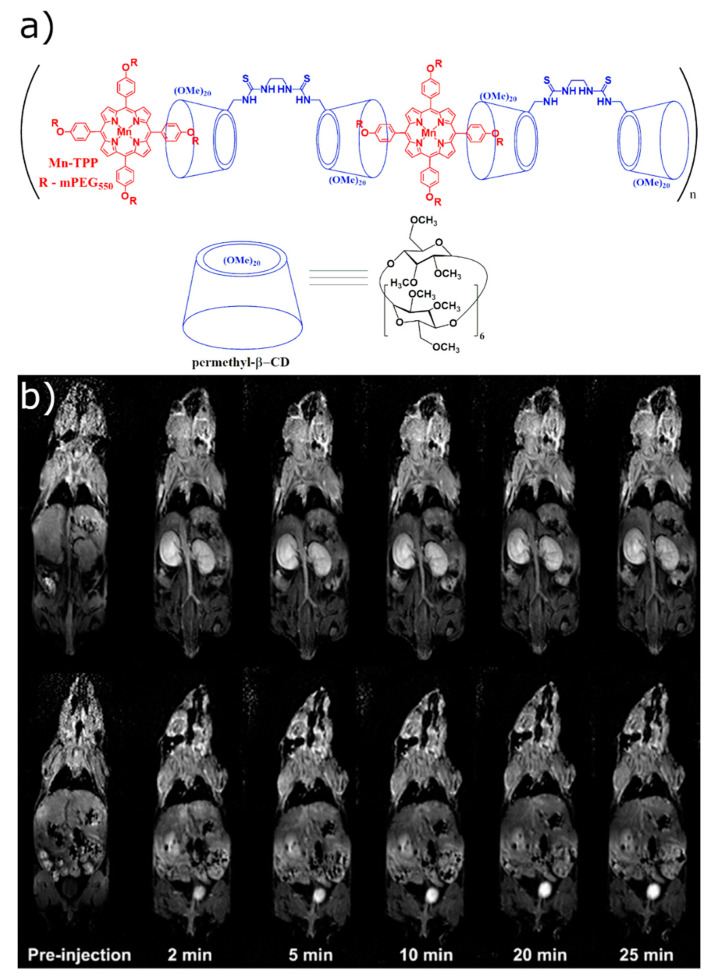
(**a**) Molecular structure of Mn(II)-TPP and bridged bis(permethyl-β-CD)s polymer. (**b**) Representative 2D coronal T1-weighted MR images of the mice at 2, 5, 10, 20, and 25 min after intravenous injection of Mn(II)-TPP/bridged β-CD magnetic resonance imaging (MRI) contrast agents at 0.03 mmol of Mn/kg [63]. The images are reprinted with permission from publisher [63].

More advanced CD-based contrast agents were demonstrated by Sun et al. who created a supramolecular complex between bridged bis(permethyl-β-cyclodextrin)s with Mn-porphyrin bearing polyethylene glycol side chains (Mn-TPP) [63]. Mice injected with this supramolecular polymer demonstrated strong contrast observed in the blood, kidneys, and bladder (Figure 2) [63]. A supramolecular polymer built using the non-covalent interaction between Mn(II)-TPP and bridged tris(permethyl-β-CD)s resulted in a longitudinal relaxivity only 7% higher compared to previously developed Mn(II)-containing linear polymer [63,64].

Work by Feng et al. conducted on CD-based NPs as MRI contrast agents demonstrated neodymium doped NaHoF_4_ NPs as T_2_ imaging agents cultured with human mesenchymal stem cells injected into the brain hemisphere of nude mice. This combination of CD-based contrast agents and stem cells [23] supports the idea of using stem cells as an MRI contrast agent carrier. Furthermore, due to the high relaxivity of the developed probe (r_2_ = 143.7 mM^−1^s^−1^ at 11.7 T) [23], use of the developed contrast agent will be beneficial for ultrahigh field MRI imaging, since the transverse relaxivity increases highly with the magnetic field strength [23,65,66].

#### 2.1.3. In Vivo Tumor Imaging

**Figure 3:**
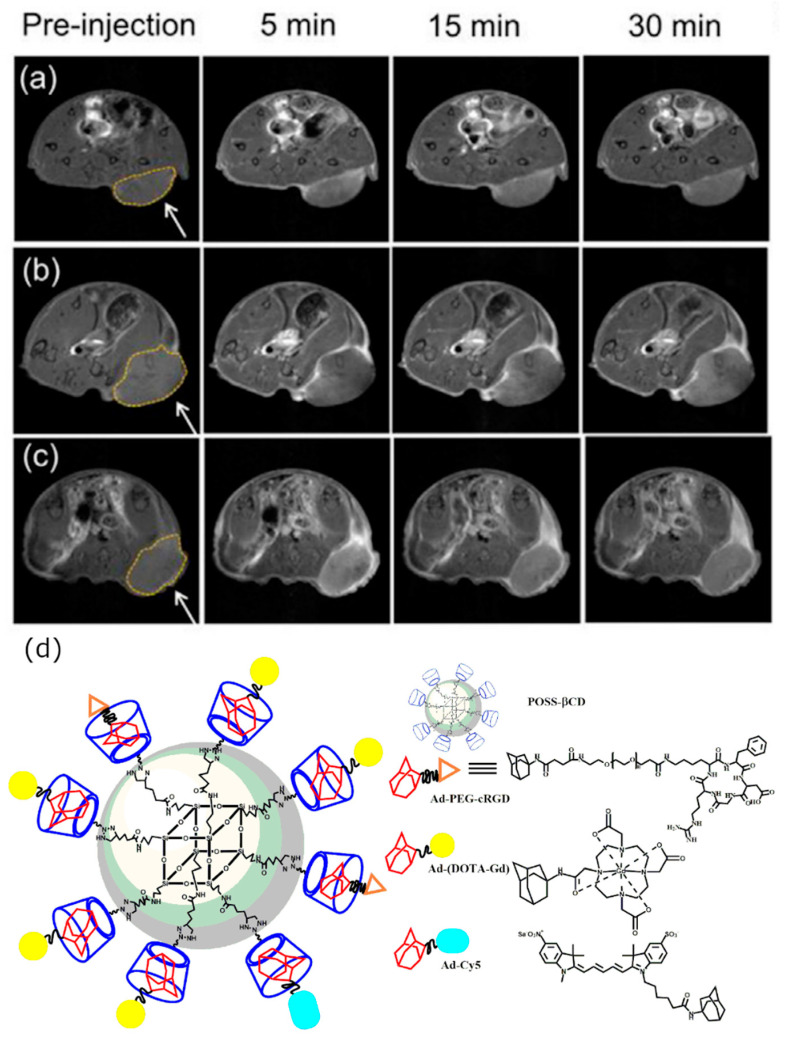
Magnetic resonance molecular imaging with cRGD-POSS-βCD-(DOTA-Gd)-Cy5 in mice bearing 4T1-Luc2-CFP tumor xenografts. The representative 2D axial fat-suppressed T1-weighted spin-echo MRI images before and at 5, 15, and 30 min post-injection of ProHance (**a**), cRAD-POSS-bCD-(DOTA-Gd)-Cy5 (**b**), and cRGD-POSS-bCD-(DOTA-Gd)-Cy5 (**c**) at 0.1 mmol-Gd/kg. The injection of cRGD-POSS-βCD-(DOTA-Gd)-Cy5 creates superior signal enhancement in tumor region. The images are reprinted with permission from publisher [67]. (**d**) Molecular structure of the developed cRGD-POSS-βCD-(DOTA-Gd)-Cy5 contrast agent.

Imaging of cancer is one of the hot topics in modern medical imaging field. Despite the numerous developed contrast agents discussed above, only Zhou et al. used multiple β-CDs attached to a polyhedral oligomeric silsesquioxane nano globule at a targeted nano globular contrast agent from host–guest assembly for magnetic resonance cancer molecular imaging [67]. The host–guest contrast agent bonds to α_v_β_3_ integrinin 4T1 malignant breast tumor through cyclic RGDfK peptide and gives greater contrast enhancement, due to the α_v_β_3_ that is overexpressed in tumors (Figure 3a–c). This designed contrast agent produced superior contrast and signal enhancement compared to the clinically used Gd-based ProHance and non-targeted control cRAD-POSS-bCD-(DOTA-Gd)-Cy5 contrast agent. Molecular structure of cRGD-POSS-βCD-(DOTA-Gd)-Cy5 imaging agent is shown on Figure 3d.

### 2.2. Direct Labeling of the CD Molecules

Another approach of synthesis of CD-based contrast agents for MRI imaging purposes is to conjugate metal–organic Gd(III)-containing complexes to the CD molecule through covalent bonds. We refer to this method as direct labeling of CD molecules. The key advantage of this approach is the availability of CD cavity for host–guest interaction with other molecules that can be effectively used for drug delivery study and imaging of molecular interactions.

Synthetically, modifying CDs in order to meet the criteria of an ideal contrast agent can be summarized by three key components: having a point of functionalization to attach the chelating group, designing a rigid linker in order to slow local movements, and lastly, conjugation of the macrocyclic complex in order to encapsulate multiple lanthanide chelates—thus, overall enhancing MRI signal, while improving overall stability and relaxivity profiles [35,54,55,56,68].

**Figure 4:**
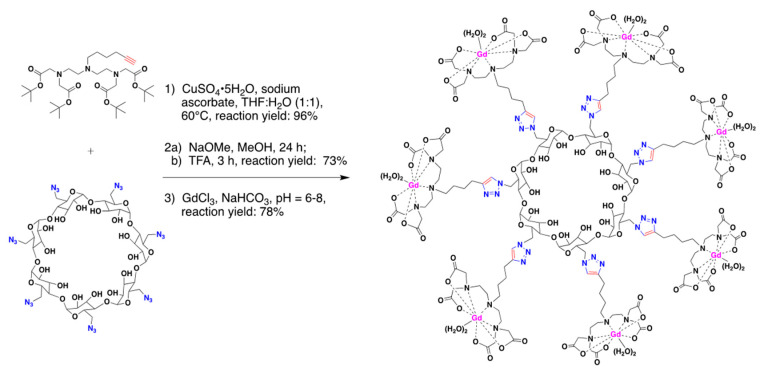
Multivalent β-CD “Click cluster”, containing seven paramagnetic chelating groups, each with two water exchange sites linked via triazole-based linkers. The β-CD “Click cluster” was synthesized from per-azido-β-CD precursor and conjugated using the well-established Huigsen cycloaddition reaction. Figure adapted from Bryson et al. with permission from publisher [35].

Various synthetic approaches have been explored, which demonstrates CD’s ability to be functionalized and conjugated in a robust fashion dependent on the desired application(s) [35,54,55,56,68]. Bryson et al. synthesized a monodisperse β-CD Click cluster containing seven paramagnetic chelates encompassing two water exchange sites [35]. Using Click chemistry, an alkyne-functionalized dendron was reacted with the per-azido-β-CD to yield the desired product (Figure 4). Using similar methods, Champagne et al. recently reported the synthesis of a different β-CD MRI probe containing seven iminodiacetate arms connected at the C6-position of β-CD by a triazole-based linker following a copper(I)-mediated 1,3-dipolar cycloaddition [54]. In addition, CDs can be differentially conjugated to produce multifunctional probes. Kotková et al. synthesized a novel bimodal fluorescence/MRI probe using a β-CD scaffold [55]. β-CD was labeled first using fluorescein isothiocyanate (FITC) and subsequentially with an isothiocyanate derivative containing a DOTA-based ligand [55]. The rigidity of the linker between the CD and the Gd-containing ligand plays an important role in increasing T_R_, thus enhancing the overall MRI signal [35,55]. Additionally, multifunctional NPs have been modified using an asymmetrically functionalized β-CD-based star copolymer by conjugating β-CD using doxorubicin (DOX), folic acid (FA), and DOTA-Gd moieties [54]. Similar to the work of Bryson et al. [35] and Champagne et al. [54], the key conjugation method used was azide-alkyne Huigsen cycloaddition, creating rigid triazole linkers.

#### 2.2.1. In Vitro Development

Skinner et al. demonstrated the first labeling of CD macrocycle with a Gd(III) chelate albeit the CD cavity was still used for non-covalent binding to another Gd(III) chelate in order to increase the proton relaxivity. [69]. Relaxivity of this complex increased when it was bound noncovalently to another gadolinium complex with the addition of two phenyl moieties.

Bryson et al. created a contrast agent with a ten-fold increase in relaxivity at 9.4 T compared to clinically available Magnevist by labeling of per-azido-β-cyclodextrin core with seven diethylenetriaminetetraacetic acid (DTTA) Gd(III) chelates [35]. This development can be attributed to the increased Gd(III) ions per molecule and further increase in relaxivity due to conjugation to the macrocycle. In addition to the high relaxivity, unoccupied cavity of β-CD makes the developed contrast agent an excellent host scaffold to functionalize through noncovalent assembly with biological receptor-specific targets.

Bimodal MRI-fluorescence probes were demonstrated by Kotkova et al. [55] who combined a DOTA-based ligand with fluorescein functionality to simultaneously obtain fluorescence and MR images. Although the developed CD-based agent was studied in vitro, the benefit of this scaffold for MRI visualization under in vivo conditions was assumed due to its low cytotoxicity and high cell uptake. Fredy et al. developed cyclodextrin polyrotaxanes as a highly modular platform for an imaging agent [70]. Selectively functionalized cyclodextrins with a Gd(III) complex or BODIPY fluorescent tag were put on to a polyammonium chain to form polyrotaxanes. From this, polyrotaxanes could be assembled with fluorescent CDs and CDs with dia- or paramagnetic lanthanide complexes. Each threaded cyclodextrin was molecularly defined, which is an advantage over statistical post-functionalization of CD-polyrotaxanes. In vitro studies demonstrated that the Gd-bearing polyrotaxanes have relaxivities that are five times higher than Gd-DOTA, which makes them effective contrast agents for MRI applications [70].

NPs fabricated using biological macromolecules have been demonstrated by both Liu et al. [68] and Su et al. [71]. Liu designed pH disintegrable β-CD-based micellar NPs, while Su reported star-like dextran wrapped superparamagnetic iron oxide NPs. Both groups reported dual effects: an imaging contrast agent and cytotoxicity to HeLa cells at high concentrations, making these molecules both imaging probes and potential chemotherapeutics agents. Later, the synthesis of the contrast agent that affects the spin-spin (T_2_) relaxation was suggested by conjugating β-CD to magnetic NPs [72].

#### 2.2.2. In Vivo Imaging of CD-Based MRI Contrast Agents Based on the Direct Labeling of CD Cavity

**Figure 5:**
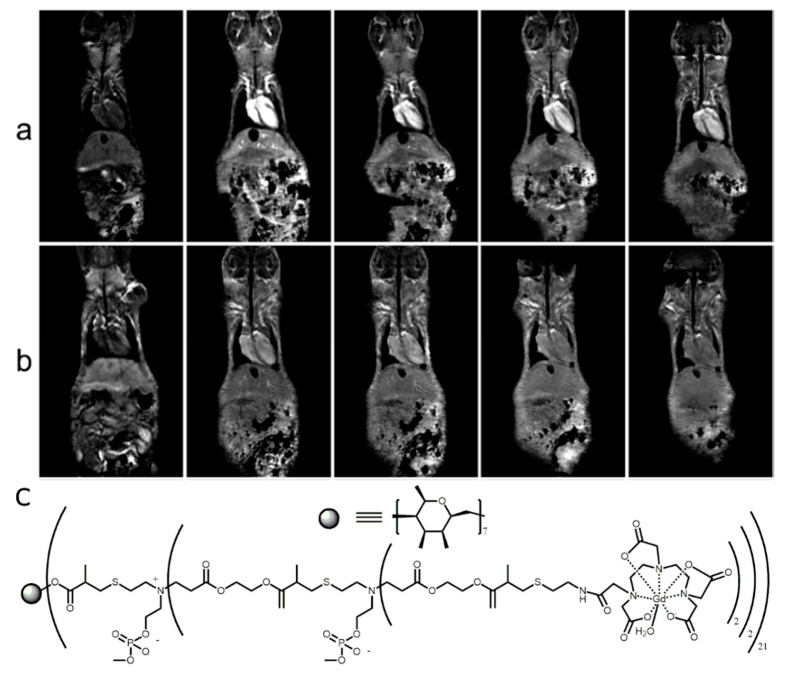
Representative T1 weighted multislice image of the mice heart after 0.1 mM Gd/kg injection of G2/MOP–DTPA–Gd contrast (**a**) and Magnevist (**b**). The superior contrast-to-noise ratio was observed from the heart after the G2/MOP–DTPA–Gd injection. The images (**a**) and (**b**) are reprinted with permission from publisher [24]. (**c**) The molecular structure of the developed G2/MOP-DTPA-Gd CD-based contrast agent.

In vitro studies have paved the way for a number of groups to demonstrate CD-based MRI contrast agents in vivo. Vascular imaging has long been a molecular imaging goal allowing investigators the ability to image the vasculature with high contrast. The G2/MOP–DTPA–Gd contrast agents synthesized from polyester dendrimers with β-CD core have been demonstrated with high yield and fast synthesis while providing for 2.7 times the relaxivity of Magnevist (DTPA-Gd) [24]. This contrast agent is rapidly hydrolyzed at the pH of 7.4 in the presence of esterase and slowly hydrolyzed at an acidic pH. The superior signal enhancement in vivo was observed following 0.1 mM Gd/kg injection (Figure 5a) and was significantly higher compared to Magnevist (Figure 5b) Furthermore, the G2/MOP–DTPA–Gd contrast agent (Figure 5c) did not show tissue retention, making the ideal blood pool and kidney imaging agent.

**Figure 6:**
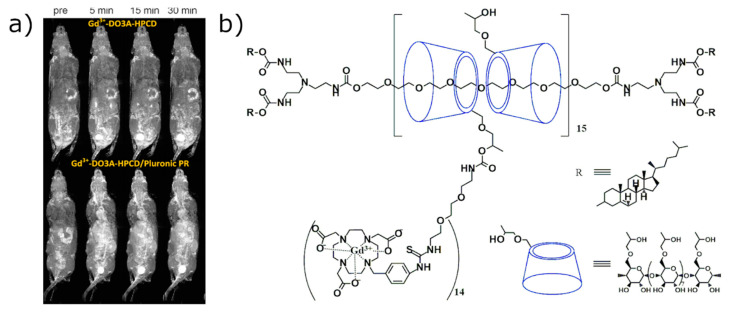
(**a**) T1 weighted 3D maximum intensity projection images of Balb/c mice. Mice were injected with Gd(III)-DO3A-HPCD (top row) or Gd(III) -DO3A-HPCD/Pluronic polirotaxane (bottom row) at a 0.03 mM-Gd/kg dose. Contrast agent distribution is shown in the images for pre injection and 5, 15, and 30 min after injection in to the tail vein with Gd3+ complexes. Images have been re-printed with permission from publisher [73]. (**b**) Molecular structure of the Gd(III)-DO3A-HPCD/Pluronic polirotaxane developed by Zhou et al.

Zhou et al. synthesized Gd(III)-1,4,7,10-Tetraazacyclododecane-1,4,7-triacetic-2-hydroxypropyl-β-CD/Pluronic polyrotaxane contrast agent [73]. Interestingly, Gd-DO3A-HPCD/Pluronic polyrotaxane construct circulated for more than 30 min in the living mouse and caused about 100-fold vascular enhancement when compared to the monomeric form (Figure 6a). Furthermore, the polyrotaxane derivative showed a much higher signal enhancement after 5 min in the heart than the monomeric form but underwent rapid elimination by renal filtration, preventing blood enhancement. Thus, the Gd(III)-DO3A-HPCD/Pluronic polyrotaxane (Figure 6b) is a promising contrast agent, which enables higher anatomic detail of blood vessel organization.

#### 2.2.3. In Vivo Imaging of Cancer

Cancer metastasis is the final insult leading to patient mortality. The early detection of metastasis is therefore a primary concern in the field of oncology. CD-based MRI contrast agents hold promise for the imaging of metastasis. Although CD-based contrast agents have yet been clinically tested, a number of groups have demonstrated these contrast agents in mammalian models.

Zhou et al. [74] reported the enhancement of MRI of liver metastases with a zwitterionized biodegradable dendritic CD-based contrast agent. Traditionally, the sensitivity in the liver for MR imaging of metastases is low due to the accumulation of the contrast agent in the Kupffer cells and hepatocytes instead of cancer cells. Zhou et al. used a novel dendritic contrast agent with β-CD core and the net size of 9 nm. The developed dendritic contrast agent reduces background signals in the liver significantly by avoiding being uptakes by hepatocytes and Kupffer cells through the zwitterionization, while increasing the signal in tumors through the enhanced permeability and retention effect. This CD-based zwitterionized dendritic contrast agent also showed shorter Gd(III) retention in all organs and tissues, because it could degrade into small fragments.

Zhang et al. developed polyethyleneimine- β-CD (PEI-β-CD) as a novel vector for carrying ferritin gene modified by alpha-fetoprotein promoter [75] to create a highly specific endogenous T_2_ contrast agent for hepatocellular carcinoma. In vitro T_2_-weighted and T_2_^*^-weighted MRI was used to examine the effect of ferritin heavy gene transfection. Zhang et al. observed the significant T_2_/T_2_^*^-induced MRI signal decay (up to 40%) from the BEL-7402 hepatocellular carcinoma cells treated with the developed PEI-β-CD/ferritin gene. Therefore, it was proposed that the ferritin gene carried by PEI- β-CD has a high potential to be used for early-stage MRI detection as an endogenous contrast agent for hepatocellular carcinoma imaging.

Gd (III) oxide NPs coated with folic acid functionalized poly (β-CD-co-pentetic acid) (Gd_2_O_3_@PCD-FA) as a biocompatible targeted nano-contrast agent was proposed by Mortezazadeh et al. [76]. Mortezazadeh et al. observed that Gd_2_O_3_@PCD-FA demonstrated significantly higher r_1_ and r_2_ relaxivities at 3T (r_1_ = 3.95 mM^−1^s^−1^; r_2_ = 4.6 mM^−1^s^−1^) than Gd(III)-DOTA. On the other hand, the measured relaxivities were lower compared to the pure Gd_2_O_3_ (r_1_ = 4.86 mM^−1^s^−1^; r_2_ = 5.97 mM^−1^s^−1^) due to the reduced water accessibility to Gd_2_O_3_ core in Gd_2_O_3_@PCD-FA. In order to study the performance of the developed NPs in vivo, the Gd_2_O_3_@PCD-FA contrast agent has been evaluated in the animal tumor model. Maximization of CNR was observed in 1h post-injection of the Gd_2_O_3_@PCD-FA contrast agent. Interestingly, Gd_2_O_3_@PCD-FA NPs demonstrated almost no cytotoxicity after 12 and 24 h administering to MCF-10A human normal breast cell lines.

Han et al. developed a hypoxia-targeting dendritic MRI contrast agent based on internally hydroxy dendrimer (IHD) with β-CD core [77]. The disturbance of the zwitterionic surface reduces unspecific cellular uptake by normal cells. In vivo imaging of an orthotopic breast tumor in mice injected with the developed contrast agent showed the maximization of CNR in 1 h post-injection. Han et al. observed CNR reaching the level of 10, remaining constant during the second hour, and slightly decaying 3 h post-injection.

### 2.3. Cyclodextrin-Based Contrast Agents for Hyperpolarized MRI

Although molecular imaging using MRI is challenging due to the lack of sensitivity of this imaging modality [78,79], progress has been made with the development of hyperpolarized (HP) MRI [79,80]. HP MRI utilizes the advantage of a metastable state with spin population excess significantly larger compared to the thermal equilibrium state [81]. Hyperpolarization of noble gases (such as ^3^He and ^129^Xe) is conducted via spin exchange optical pumping (SEOP), whereas polarization of ^13^C or ^1^H containing molecules is created through dynamic nuclear polarization (DNP) [82,83,84,85].

#### 2.3.1. Hyperpolarized ^13^C CD-Based Contrast Agents

**Figure 7:**
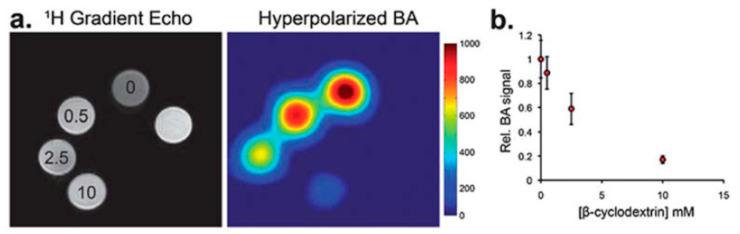
In vitro experiment at 14T demonstrating the potential application of β-CD as a contrast agent for hyperpolarized (HP) ^13^C MRI. (**a**) Proton gradient echo image demonstrating the position of phantoms with a different concentration of β-CD (0–10 mM). The HP ^13^C imaging was performed after the administration of 2.5 mM HP [1-13C] benzoic acid. It can be seen that the MRI signal decreases with β-CD concentration. (**b**) Relative MRI ^13^C signal dependence on β-CD concentration [86]. The images are reprinted with permission from publisher [86].

Due to the high signal enhancement, molecular imaging using HP ^13^C-containing molecule becomes possible. β-CD was used to create a contrast for HP ^13^C MRI [86]. Keshari et al. showed that the host–guest interaction between a β-CD cavity and HP benzoic acid substantially decrease the T_1_ relaxation of both HP ^13^C nuclei resulting in negative image contrast (decrease in signal intensity) induced by the presence of supramolecular cage (Figure 7). Based on this result, the authors proposed that similar mechanism of the negative contrast can be used to study the interaction of ligand–receptor pairs in vivo.

DNP was used to create a CD contrast agent for DNP HP MRI [87]. Caracciolo et al. observed the polarization level of 10%. Unfortunately, the T_1_ relaxation of β-CD protons was equal to 1s at 300 K, which made HP β-CD inapplicable for molecular imaging purposes. On the other hand, HP β-CD can be of interest in the fields, which require the production of strong ^1^H NMR signal from CD molecules [87]. Following this work was the hyperpolarization of methylated β-CD [88]. The methylation has been conducted using ^13^CH_3_I, which enriched the potential contrast agent with ^13^C and ^1^H nuclei. Methylated β-CDs underwent DNP and polarization levels of 7.5 and 7% were achieved for ^1^H and ^13^C, respectively. The proton T_1_ relaxation times were found to be similar to those published in [87]; however, the T_1_ relaxation time of ^13^C nuclei was equal to 3.3 and 4.9 s for fully methylated β-CDs and partially methylated β-CDs, respectively. These relaxation times allow further application of HP β-CD as contrast agents in the molecular imaging field. In addition, authors demonstrated the method of further increasing relaxation times [88].

#### 2.3.2. CD-Based Molecular Probes for Hyperpolarized ^129^Xe MRI

Current studies of molecular imaging with HP ^129^Xe MRI utilizes hyperpolarized chemical exchange saturation transfer (HyperCEST) [89,90]. The HyperCEST effect relies on a constant chemical exchange between the dissolved HP ^129^Xe nuclei in the solution and supramolecular host that can effectively encapsulate ^129^Xe [79]. Following selective depolarization of the ^129^Xe nuclei encapsulated in the supramolecular cage, the decrease in the dissolved phase ^129^Xe MRI signal can be observed as a result of the exchange dynamic [79,89].

The interaction between HP ^129^Xe and the α-CD cavity was studied for the first time in 1997 [91]. Authors observed that spin polarization induced nuclear Overhauser effect (SPINOE) and related transfer of nuclear polarization to the α-CD protons [91]. However, this approach did not become widely used in the field of molecular imaging using HP ^129^Xe.

CD-based contrast agents for HyperCEST molecular imaging became of interest recently. The first detection of HyperCEST effect using α-CD-based molecules was achieved from the α-CD pseudorotaxane complex with five carbon diethylimidazolium bar in aqueous solution [92]. The observed HyperCEST depletion was equal to 30%, which is significantly smaller compared to other molecular imaging probes [89,93]. The first β-CD-based molecular imaging probe for HyperCEST detection has been developed recently [94]. The HyperCEST contrast agent was realized as cucurbit[6]uril-based rotaxane in which β-CDs played the role of a stopper. Although rotaxane interaction with ^129^Xe was through cucurbit[6]uril cavity and no HyperCEST effect was observed from β-CDs, the presence of β-CDs was required in order to increase the solubility of the end groups [94].

**Figure 8:**
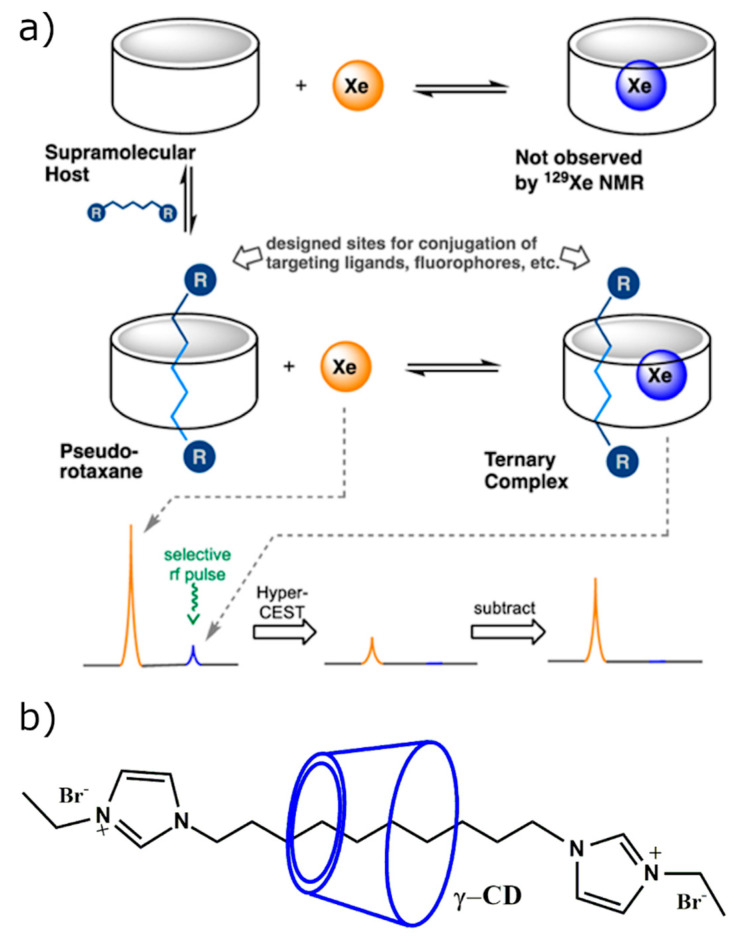
(**a**) Schematic representation of how CD-based ternary complexes are formed in the presence of HP ^129^Xe. The guest is threaded through the hydrophobic cavity of cyclodextrin and HP ^129^Xe is introduced. Detection via HyperCEST is obtained in order to determine if HP ^129^Xe is bound in the cavity of CD. The images are reprinted with permission from publisher [95]. (**b**) The developed cyclodextrin-based pseudorotaxane used for in vitro HyperCEST detection [95].

Finally, the HyperCEST effect from γ-CD-based pseudorotaxane was observed after the complexation of γ-CD with bisimidazolium guest [95]. Although γ-CD cavity is too large to sufficiently interact with HP ^129^Xe, the cavity size can be decreased by threading it with a long alkyl chain. Threading these guest molecules through the cavity of γ-CD reduces the cavity size in order for adequate HP ^129^Xe binding to occur, thus making it suitable for HyperCEST detection (Figure 8a). Potentially, this same concept can be applied for any supramolecular cage with a reasonably large cavity. The HyperCEST effect detected from γ-CD-based pseudorotaxanes was equal to 47.5% on average, which makes them interesting candidates for further application in vivo [95]. The main advantage of the HyperCEST contrast agents based on pseudorotaxane architecture over the other studied HP ^129^Xe hosts is the ease synthesis and of functionalization of the pseudorotaxanes.

In addition, γ-CD pseudorotaxane complexes prove to be a sufficient paradigm for HP ^129^Xe MRI. The synthesis of the threads proceeds in one step and can be functionalized with different terminal end groups as well as different chain lengths. The 8- and 10-carbon alkyl chains were functionalized with ethylimidazoliums (Figure 8b), strategically used to enhance water solubility of the inherently hydrophobic alkyl chain [95]. The γ-CD pseudorotaxane was comparable to that of CB6, a known xenon cage that has been responsive to in vivo HP ^129^Xe MRI [80]. Similar to CB6, the maximum HyperCEST depletion for γ-CD pseudorotaxanes was present when the samples were irradiated at a frequency of +128 ppm [95]. γ-CD pseudorotaxanes exhibited binding on the order of 10^3^ at 1:1 host–guest complexation, proving to be sufficient association for HP ^129^Xe. In addition, binding proved to be two magnitudes of order lower in fetal bovine serum, indicating that this system would work sufficiently as a potential biosensor in vivo [95].

## 3. CDs-Based Contrast Agent for Ultrasound Imaging and Photoacoustic Imaging

In addition to MRI contrast agents, CDs have been used for ultrasound (US) imaging. The first approach of synthetizing the potential contrast agent was developed by Cavalieri et al., in 2006 [96]. Air-filled polymer microbubbles functionalized with β-CDs were used as a source for contrast. The contrast originates from significant difference between acoustic impedance of tissue and air encapsulated inside of a microbubble. Cavalieri et al. found that conjugating microbubbles to β-CD preserves them from random coil to α- helix conformation transition. In addition, due to the presence of β-CD, these contrasts allow hosting molecules with hydrophobic features [96].

After nearly a decade of inactivity, the second attempt of using a β-CD-based contrast agent for US imaging was made in 2015 [44]. The authors demonstrated use of a perfluorinated FC-77/ β-CD complex. A visible blurring of signal from FC-77/β-CD caused by a disruption of the inclusion complexes under US was detected [44].

**Figure 9:**
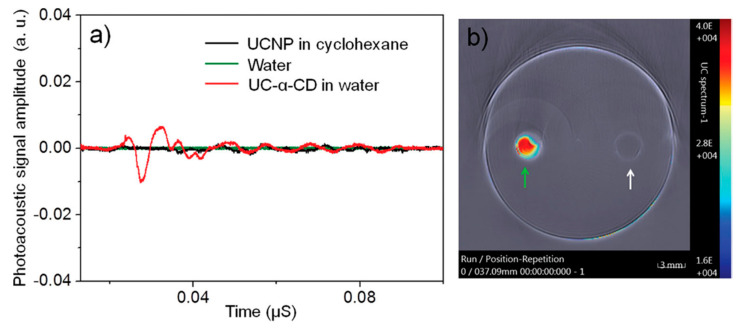
(**a**) High-resolution PA signal originated from upconversional NPs (UCNPs) in cyclohexane (black curve), distilled water (green curve), and α-CD/UCNPs (red curve) in water. The excitation was conducted using 980 nm nanosecond pulsed laser. (**b**) Photo-acoustic imaging (PAI) of tissue-mimicking phantom containing chambers filled with α-CD/UCN in water (green arrow) and distilled water (white arrow) [49]. The images are reprinted with permission from the publisher [49].

Another US medical imaging modality that utilized CD-based contrast agents was PAI. The first CD-based contrast agent was synthetized by surface modification of oleic-acid (OA) stabilized upconversional NPs (UCNPs) NaYF_4_:Yb^3+^, Er^3+^ with α-CD [49]. α-CD formed an inclusion complexes with an OA yielding to luminescence quenching of UCNPs and production of a strong PA signal instead (Figure 9a). Cytotoxicity studies demonstrated no toxicity of α-CD/UCNPs contrast agents. Following in vitro imaging (Figure 9b), the first PAI in a living mouse was conducted using 980 nm excitation laser. These results demonstrated the ability of α-CD/UCNPs to be an efficient PAI contrast agent for diagnostic purposes [49].

An effective PAI agent for imaging prosthetic joint infection (PJI) [48] was demonstrated by conjugation of β-CD to indocyanine green (ICG). The β-CD-ICG PAI agent was demonstrated in the mice model of PJI. Wang et al. found that conjugation of ICG to β-CD improves its PA signal generation. Although the PAI signal increase was not significant with β-CD-ICG contrast, it still demonstrated the ability to serve as a contrast agent for non-invasive diagnostic of PJI [48].

Yu et al. recently developed a CD-based PAI contrast agent sensitive to tumor environment [47]. Gold NPs (AuNP) were modified initially with pyridine-2-imine-terminated single stand DNA via gold-thiol bonds, and α-CDs were capped on the end of DNA through hydrophobic interaction with CD’s cavity. The α-CD-AuNP agent produced no PA signal under neutral pH conditions, but upon entering the tumor, α-CDs separate from the DNA ends due to reduction in non-covalent forces. This study demonstrated that a developed α-CD-AuNP contrast agent can be successfully used as a tumor-selective theranostic agent [47].

## 4. Radiolabeled CD-Based Contrast Agents

PET and SPECT are imaging modalities that require probe radiolabeling to produce tomographic images. These modalities have superior sensitivity and deeper tissue penetration compared with luminescence-based imaging and MRI [97]; they require lower dose of imaging agent for functioning. However, the application of PET/SPECT tracers is limited to the half-life of radiolabeled isotopes and biodistribution in the living organism.

The first demonstration of the CD-based PET molecular imaging probe was done by Bartlett et al., in 2007 [25]. CD-containing NPs were studied as delivery agents for transferrin (Tf)-targeted delivery to tumors of siRNA molecules. Nontargeted and Tf-targeted siRNA NPs were synthetized by using cyclodextrin-containing polycation. One percent of the targeted NPs containing adamantane-PEG molecules on the surface were modified with Tf. Si-RNA were conjugated with DOTA to the 5′ end with further ^64^Cu labeling [25]. MicroPET revealed negligible impact of the attachment of the Tf targeting ligand to the NPs on biodistribution. Unfortunately, nearly identical tumor localization kinetics of both targeted and nontargeted ^64^Cu-DOTA-siRNA NPs were observed; tumor accumulation was also similar at 1 day after injection (≈1% ID/cm^3^). However, bioluminescent imaging showed the intracellular localization and functional activity of siRNA delivered by Tf-targeted NPs in the tumor cells 1 day after injection.

Interestingly, the presence of CD in therapeutic compounds allows radio isotopic labelling for PET imaging of the drug circulation. In vivo biodistribution of the IT-101, clinically developed drug for cancer treatment, in mice with Neuro2A tumors was studied by Schluep et al. [98]. IT-101 contains molecule drug camptothecin (CPT) conjugated with β-CD based polymers (CDP), which acts as a carrier system for active molecule. The CDP site was labeled with ^64^Cu through attaching of DOTA complex for microPET imaging; the obtained nanoparticle was similar to the IT-101 structure. Plasma pharmacokinetics of ^64^Cu-IT-101 ere studied at 1, 4, and 24 h after injection with microPET/CT. It was shown that a low-molecular-weight fraction cleared rapidly through kidneys due to biphasic elimination profile, whereas remaining NPs circulated with a terminal half-life of ≈13 h. Biodistribution of IT-101 was examined at 24 h after administration; the highest tissue concentration was found in the tumor followed by the liver.

Another study conducted to investigate the effect of CD inclusion in NPs for drug delivery revealed the possibility of CD-based SPECT imaging agent [42]. Areses et al. compared the adhesive abilities and biodistribution of orally administered poly(anhydride) NPs and CD containing NP (CD-NP) in rats utilizing labeling with ^99m^Tc for SPECT imaging. ^99m^Tc-NP showed activity only in the gastrointestinal tract on SPECT images, whereas ^99m^Tc-CD-NP revealed extended residence time in stomach: about 13% of ^99m^Tc-CD-NP administered dose and 3% of ^99m^Tc-NP given dose were found in the stomach after 8 h.

Liu et al., in 2011 [39], studied the improvement of the biodistribution of NPs using CD. Rare-earth UCNPs were modified by α-CD and OA for increasing of the water-solubility. UCNP-OA-CD complexes with Tm inclusion were labeled with ^18^F (^18^F-UCNP(Tm)-OA-CD) for microPET imaging of ex vivo and in vivo biodistribution in mice at 5 min and 2 h. This was the first labeling of CD-based probe with an ^18^F. Ex vivo imaging displayed rapid accumulation of NPs in the liver (~90.8% injected dose(ID)/g) and spleen (~62.5% ID/g) at 5 min and further decreasing of liver uptake to ~57.6% ID/g, while increased spleen accumulation to 118.9% ID/g after 2 h post-injection. In vivo microPET images were consistent with ex vivo biodistribution results and showed intense radioactive signals in the liver and spleen at 5 min after injection.

**Figure 10:**
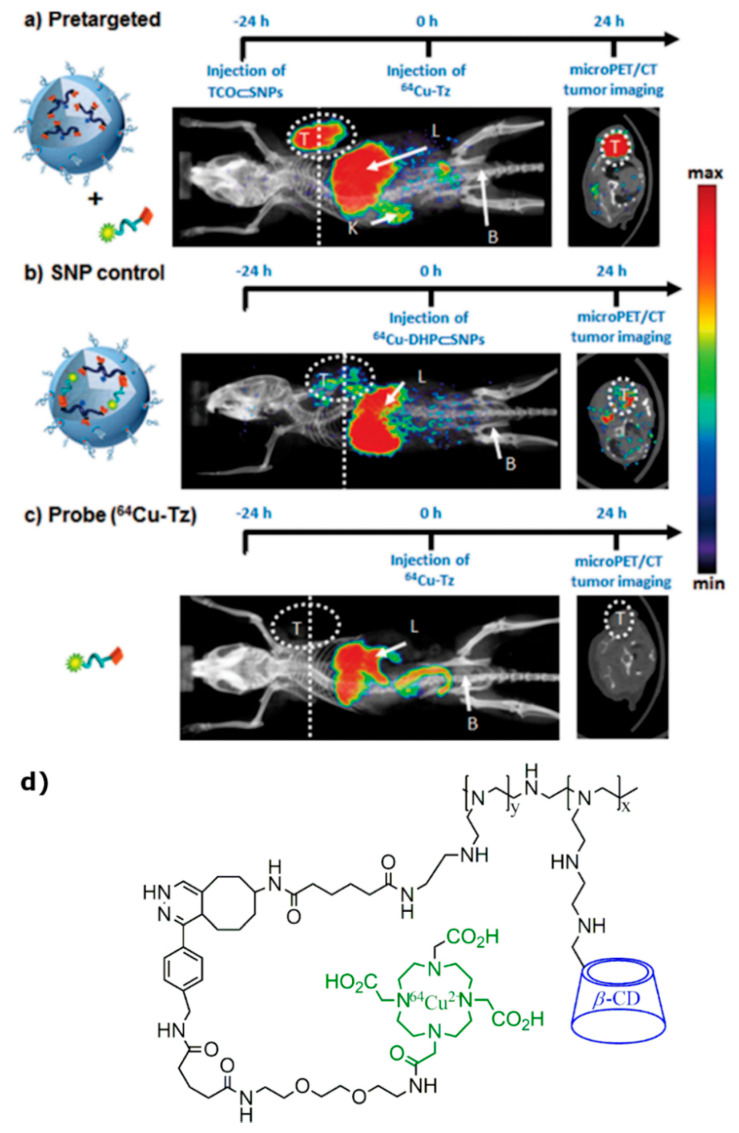
Timeline of the injection protocol employed for (**a**) pre-targeted, (**b**) SNP control (64Cu-DHP⊂SNPs), and (**c**) free radiolabeled reporter (64Cu-Tz) studies. Representative in vivo microPET/CT images of the mice (n = 4/group) subjected to the three studies at 24 h p.i. Labels T, L, K, and B refer to the tumor, liver, kidney, and bladder, respectively. Dashed lines correspond to the transverse cross-section through the center of each tumor mass, whose image is shown in the right panel [99]. The images are reprinted with permission from publisher [99]. (**d**) The chemical structure of the tumor targeting imaging probe developed by Hou et al. [99].

In addition to the previously discussed reports, a pre-targeted approach for molecular imaging probe development was presented by Hou et al. [99]. This work was based on biorthogonal conjugation chemistry between NPs, which have tendency to accumulate in tumors to enhanced permeability and retention (EPR) effect and radiolabeled imaging agents for PET imaging. One of NPs components was synthesized from CD-grafted polyethyleneimine (CD-PEI) and trans-cyclooctene N-hydroxysuccinimide (TCO-NHS), resulted in TCO/CD-PEI building block. Actual tumor-targeting NP (TCO⸦SNPs) was prepared via self-assembly from four different blocks TCO/CD-PEI, CD-PEI, adamantane-grafted polyamidoamine (Ad-PAMAM), and Ad-grafted polyethylene glycol (Ad-PEG) and injected to the tail vein of the mice with U87 glioblastoma cells. After EPR-driven accumulation in tumor, TCO⸦SNPs can dynamically disassemble to release TCO/CD-PEI. After 24 h post-injection of TCO⸦SNPs, freshly prepared tetrazine compound radiolabeled with ^64^Cu through DOTA (^64^Cu-Tz) was injected. Subsequently, distributed ^64^Cu-Tz can undergo biorthogonal reaction with TCO/CD-PEI parts left after TCO⸦SNPs disassembling in vivo, yielding the dihydropyrazine conjugation adduct ^64^Cu-DHP/CD-PEI, which acts as a contrast agent for tumors. Multiple microPET and anatomical CT images were acquired following the injection of pre-targeted NPs and ^64^Cu-Tz compound with in vivo reaction (Figure 10a), along with two series of control PET imaging of a fully ex vivo prepared ^64^Cu-DHP/CD-PEI adduct (Figure 10b) and free radiolabeled reporter ^64^Cu-Tz (Figure 10c). Pre-targeted studies showed the accumulation and retention of radioactivity mainly in the glioblastoma tumor and liver and some nonspecific uptake by tissues. Supramolecular nanoparticles (SNP) control and probe (^64^Cu-Tz) imaging did not present highly distinguishable tumor uptake. Although, high radioactivity was observed in the liver in all three cases, which can be explained by ^64^Cu^2+^ dissociation from DOTA ligand. This issue can be eliminated by using different radioisotopes for labelling.

Modification of CD by grafting alkyl chains (C6-C14) can lead to self-organization of obtained derivatives into NPs potentially useful for drug delivery [100]. Further co-nanoprecipitation of bio-esterified alkylated cyclodextrins with PEGylated phospholipids (PEG) can lead to surface-modified NPs [101]. Perret et al. researched the effect of the PEG chain length on the plasma protein absorptivity and blood kinetics of NPs [43]. β-CD derivatives with C10 alkyl chain (β-CD-C10) were co-nanoprecipitated with PEG with chain length of 2000 (^125^I-βCD-C10-PEG_2000_-NP) and 5000 Da (^125^I-βCD-C10-PEG_5000_-NP) and radiolabeled with ^125^I for SPECT ex vivo and in vivo biodistribution studies. In vivo SPECT/CT images were acquired at 10 min, 1, 3, 6, and 24h following the injection of NPs without PEG (^125^I-βCD-C10-NP) and with PEG (^125^I-βCD-C10-PEG_2000_-NP, ^125^I-βCD-C10-PEG_5000_-NP). Hepatic activity was observed with all NPs; however, splanchnic activity was observed only with ^125^I-βCD-C10-NP. Additionally, ^125^I-βCD-C10-PEG_5000_-NP systems showed reduced elimination and increased circulating concentration following in vivo intravenous injection in comparison with other NPs.

β-CD-based rotaxane were used in developing theranostic shell-crosslinked NPs (SCNPs) by Yu et al. for improving drug delivery and controllable release in supramolecular medicine [102]. The core-shell-structured self-assembling NPs were obtained from polyrotaxanes consisted of amphiphilic diblock copolymer and the primary-amino-containing β-CD (β-CD-NH_2_), which undergoes complexation with poly(ε-caprolactone) (PCL) segment. In the gained structure, amphiphilic deblock copolymer acts as the axle, and β-CD-NH_2_ acts as a wheel in complex with PCL, whose chains can experience hydrophobic interactions along with the perylene diimide (PDI) stoppers, which has a tendency to π-π stacking interactions. Obtained SCNPs were labeled with radioactive ^64^Cu through DOTA attachment to SCNPs (^64^Cu SCNPs@DOTA) for PET imaging of the dynamic biodistributions and accumulations of SCNPs in the main organs. HeLa tumor-bearing mice were imaged at various time-points after intra-venous injection 150 μCi of ^64^Cu SCNPs@DOTA. Images revealed the high liver uptake of ^64^Cu SCNPs@DOTA along with increasing of tumor uptake from the point of injection to 12 h post-injection with further start of clearance at 48 h.

Another study utilized PET for investigation in vivo distribution of 2-Hydroxypropyl-β-CD (HPBCD), β-cyclodextrin derivative, and an orphan drug for the Niemann–Pick disease treatment [40]. Six-deoxy-6-monoamino-(2-Hydroxypropyl)-β-CD (NH2-HPBCD) was conjugated with p-NCS-benzyl-NODA-GA (NODAGA) and radiolabeled with ^68^Ga for PET/CT imaging. Ex vivo and in vivo studies on healthy mice showed that ^68^Ga-NODAGA-HPBCD was mainly excreted through the urinary system with low uptake of the abdominal and thoracic organs and tissues at 30 and 90 min post-injection.

The most recent study in the area of CD-based PET molecular imaging probes containing tumor targeting compounds was done by Trencsenyi et al., in 2019 [41]. Their aims were to develop novel radiolabeled compound specific to the prostaglandin E2 (PGE2), which plays an important role in tumor progress and formation of metastases. The high affinity of PGE2 to the randomly methylated β-CD (RAMEB) was reported by Sauer et al. [103]. Trencsenyi et al., in their research, aimed to synthesize PGA-specific RAMEB labeled with ^68^Ga through NODAGA (^68^Ga-NODAGA-RAMEB) for investigation of its tumor-targeting properties and in vivo biodistribution using PET. PancTu-1 and BxPC3 tumor-bearing SCID mice were intravenously injected with ^68^Ga-NODAGA-RAMEB. The injection was followed with dynamic and static microPET imaging at 0–90 min. The accumulation of ^68^Ga-NODAGA-RAMEB was significantly higher in BxPC3 tumors than in the PancTu-1; the highest post-injection tumor-background ratio (T/M) was obtained at 80–90 min post-injection. The T/M standardized uptake values (SUVs) were 10-fold lower in the PancTu-1 than those of BxPC3 tumors confirming the high PGE2 selectivity of ^68^Ga-lebeled cyclodextrin.

## 5. CD-Based CT Contrast Agents

**Figure 11:**
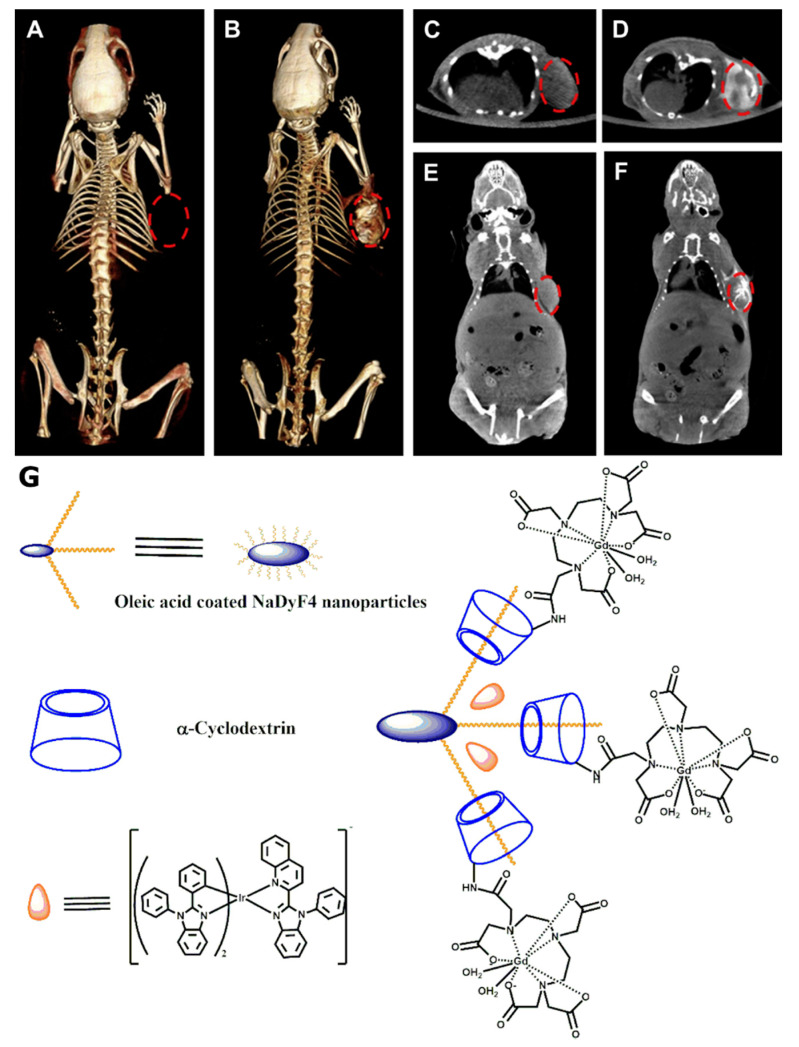
In vivo 3D volume-rendered (**A**,**B**) and maximum intensity projections in axial (**C**,**D**) and coronal (**E**,**F**) view CT images of the tumor-bearing mouse obtained pre- (**A**,**C**,**E**) and post- (**B**,**D**,**F**) injection of Ir-Gd-α-CD-DyNPs contrast agent [53]. The images are reprinted with permission from the publisher [53]. The position of the tumor was marked by red circles. The chemical structure of the developed CT contrast agent is shown in (**G**).

The initial development of CD-based contrast agents for CT was used to investigate multimodality imaging potential of phosphorescent-modified NaDyF_4_ NPs (DyNPs) [53]. OA-coated DyNPs underwent complexation with α-CD followed by conjugation with Gd(III)-DTPA complex. The last stage of contrast agent formation was the loading of phosphorescent Ir(Dbz)_2_(Pbi) complex with in the hydrophobic layer of Gd-α-CD-DyNPs [53]. Besides the contrast creation potential for fluorescence and MRI, Zhou et al. considered the developed Ir-Gd-α-CD-DyNPs as a potential contrast agent for CT due to the presence of heavy atoms of Dy, Gd, and Ir. The measured Hounsfield units (HU) value of 10 mg/mL Ir-Gd-α-CD-DyNPs aqueous solution was equal to 158 at 80 kV X-ray energies. Following in vitro experiments, Ir-Gd-α-CD-DyNPs were used for CT scanning of tumor-bearing mice (Figure 11). The original HU value of 109 for the tumor increased to 212 HU after intratumoral injection of 100 ul of 3 mg/mL Ir-Gd-α-CD-DyNPs (Figure 11G) solution. As a result, approximately 100% signal enhancement was observed on the CT scans.

Another multimodal CD-based contrast agent that was developed for CT scanning was a core-shell-structured alkali ion-doped CaF_2_:Yb,Er UCNP [50]. Similar to the NPs discussed above, the core of NPs was coated with OA with subsequent complexation with α-CD. The final α-CD/UCNPs contrast agent was compared to well-known iopromide 300 CT clinical contrast agent. Yin et al. found that developed α-CD/UCNPs have 45% higher HU value at concentration 80 mM/L. This high X-ray absorption originates from the present high atomic number elements. In addition, investigated α-CD/UCNPs were loaded with doxorubicin, and the ability of simultaneous cancer imaging and treatment using this contrast agent was suggested [50].

The next achievement in the CT contrast agent development was obtained in 2017 by conjugating β-CD to poly(methyl vinyl ether-alt-maleic anhydride) (PVME-alt-MAH) with a subsequent reaction using modified dextran to create gel microspheres [104]. To make dextran microspheres visible for CT, they were loaded with iodine in *n*-hexane. Through the micro-CT phantom imaging, Zhu et al. observed the subsequent improvement of contrast after iodine loading.

Another study, conducted in 2017, demonstrated synthesis of β-CD-{poly(ε-caprolactone)-poly(2-aminoethyl methacrylate)-poly[poly(ethylene glycol) methyl ether methacrylate]}_21_ (β-CD-(PCL-PAEMA-PPEGMA)_21_) with stable unimolecular micelles formed in aqueous solution [51]. β-CD-(PCL-PAEMA-PPEGMA)_21_ had used a template for the creation of gold NPs (AuNPs) with uniform sizes, followed by the encapsulation of doxorubicin (DOX). The CT performance of the final β-CD-(PCL-PAEMA-PPEGMA)_21_/AuNPs/DOX contrast agent was compared to the clinically available Omnipaque both in vitro and in vivo. At a concentration of 800 uM, Lin et al. found the X-ray absorption of β-CD-(PCL-PAEMA-PPEGMA)_21_/AuNPs/DOX solution to be approximately 23% higher compared to Omnipaque. In vivo imaging of β-CD-(PCL-PAEMA-PPEGMA)_21_/AuNPs/DOX contrast agent in HepG2 mice tumor model demonstrated significant enhancement of CT signal compared to the clinical iodine analogue [51].

Another step in the development of CD-based CT contrast agents was done by creation an unimolecular micelle system synthesized from 21-arm star-like polymer β-CD-{poly(lactide)-poly(2-(dimethylamino) ethyl methacry late)-poly[oligo(2-ethyl-2-oxazoline)methacrylate]}_21_ (β-CD-(PLA-PDMAEMA-PEtOxMA)_21_) followed by production of β-CD-(PLA-PDMAEMA-PEtOxMA)_21_/AuNPs/DOX [105]. The developed Au-loaded β-CD-based contrast agent was tested in the animal HepG2 tumor model. Lin et al. found that intravenously injected β-CD-(PLA-PDMAEMA-PEtOxMA)_21_/AuNPs/DOX produces substantially higher CT contrast compared to iodine-based Omnipaque, which was used for control scans.

Following the previous studies, the most recent advance in the field of CD-based CT contrast agents was achieved by the same group [106]. The authors synthetized 21-arm star-like polymers β-CD-g-{poly(2-(dimethylamino)ethyl methacrylate)-poly(2-hydroxyethyl methacrylate)-poly[poly(ethylene glycol) methyl ether methacrylate]} (β-CD-g-(PDMA-b-PHEMA-b-PPEGMA)). By adding HAuCl_4_ solution into β-CD-g-(PDMA-b-PHEMA-b-PPEGMA) aqueous solution and triggering subsequent reduction with DMA, the AuNPs at the core of unimolecular micelles were formed. The CT contrast of β-CD-g-(PDMA-b-PHEMA-b-PPEGMA)/AuNPs agent was compared to contrast created by Omnipaque in vitro. Lin et al. found that, at a concentration of 800 uM, the X-ray attenuation of β-CD-g-(PDMA-b-PHEMA-b-PPEGMA)/AuNPs was approximately 37% bigger than that of Omnipaque. The developed β-CD-g-(PDMA-b-PHEMA-b-PPEGMA)/AuNPs demonstrated slightly higher X-ray absorption compared to previously synthetized β-CD-(PCL-PAEMA-PPEGMA)_21_/AuNPs [51,106]. However, the average HU values of β-CD-g-(PDMA-b-PHEMA-b-PPEGMA)/AuNPs at each concentration were slightly lower than previously developed β-CD-g-(PLA-b- PDMA-b-PEtOxMA)_21_/AuNPs [105,106].

## 6. Discussion

Starting in the early 1990s, CDs became of large interest for developing contrast agents and molecular probes for medical imaging. Although the primary application of CDs in medical imaging was the basis for developing novel Gd-based MRI contrast agents, CD-based contrast agents commence to be utilized by other imaging modalities such as PET, CT, US, and PAI. This growing interest is caused by excellent biocompatibility, low toxicity, and relative ease of modifying of CD molecules. Currently, the most frequently used CD molecule for contrast agent development is β-CD. Despite the extensive development of β-CD-based contrast agents, some of the medical imaging areas started utilizing α- and γ- CD-based contrast agents and molecular probes as well.

The working mechanism of the vast majority of CD-based contrast agents for conventional MRI relies on the Freed’s theory [57] and the decreasing of the relaxation times of the solvent protons once either paramagnetic ion or metal–organic complex containing the paramagnetic ion binds to a supramolecular cage. Initially, this binding was done through hydrophobic interaction between CDs cavity and metal–organic complexes. Although contrast agents synthesized using this approach demonstrated significantly better performance compared to the clinically available analogs, the main disadvantage of this technique is blocking the supramolecular cage cavity, which makes the developed contrast agents and molecular probes uncapable to image some drug transport, biodistribution, and treatment monitoring. In addition, the number of paramagnetic ions per molecule is also limited, since usually only one metal–organic complex enters the cavity. This issue was overcome by creating different CD-based polymers and dendrimers, and the number of macromolecules determined the maximum number of paramagnetic ions per molecule. Starting from the early 2000s, the direct labeling of the CD macrocycles with chelates containing paramagnetic ions has been developed intensively. This method allows keeping the CD cavity free for interactions with different molecules. Therefore, the contrast agents based on the direct labeling of the CD macromolecules have a significantly larger field of applications compared to agents developed using non-covalent interactions.

Currently, CD-based MRI contrast agents contain mostly Gd(III) ions and are designed as T_1_ contrast agents. On the other hand, the development of CD-based T_2_ and T_2_^*^ contrast agents have been demonstrated in vitro and in vivo [23,71,75]. Although some of the developed CD-based contrast agents for proton MRI demonstrated an overall low level of cytotoxicity, dedicated toxicology studies are needed prior to the further translation of this imaging agents into clinics. In addition, the vast majority of the relaxivity measurements were conducted at low magnetic fields, which are not for clinical imaging purposes, such as 0.47 and 0.5 T. The studies that focused on animal imaging were mostly conducted at high magnetic fields, such as 7 and 9.4 T. In order to facilitate the potential clinical transition of the developed CD-based contrast agents, the accurate measurement of relaxivities and comparisons to clinical analogs should be performed at 1.5 and 3 T, which are currently used for clinical MRI. During the last eight years, CDs drew attention from researchers working with hyperpolarized MRI. Although only a few studies were conducted, the obtained results demonstrated the high potential of CD macromolecules to become a basis for further development of hyperpolarized probes. Recently, Hane et al. demonstrated that γ-CD-based pseudorotaxanes can be a valuable platform for developing the molecular imaging probes that utilizes the HyperCEST effect [95]. The alkyne chains have a high affinity to the γ-CD cavity and can be easily designed to serve as a high-affinity probe that has a selective binding to the disease site. Functionalizing the alkyne chain should be done carefully such that there is no effect on the interaction with hyperpolarized ^129^Xe. The HyperCEST depletion observed was only around 50% indicating partial depolarization of hyperpolarized ^129^Xe encapsulated by γ-CD-based pseudorotaxanes. An accurate study for radiofrequency saturation pulses is required to maximize the HyperCEST depletion and to translate γ-CD-based pseudorotaxanes to in vivo imaging applications. Although the demonstrated results of hyperpolarized MRI molecular imaging probes look promising, further in vivo imaging studies are needed, as well as biodistribution and toxicity evaluations of the proposed biosensors.

Following the success of the CD-based MRI contrast agents, several attempts have been made to use CDs as a contrast agent for US imaging [44,96]. The developed US CD-based contrast agents caused significant acoustic impedance difference between the tissue and the contrast agents. Despite successful in vitro demonstration of this proof-of-principle [44], there was no further development of the CD-based contrast agent for US imaging purposes during the last five years. On the contrary, CD-coated NPs became of interest for the PAI. It was found that coating of UCNPs with α-CD cause photoacoustic signal enhancement [49] and, therefore, can serve as efficient PAI contrast. The most recent achievement in the field demonstrated PAI detection of prosthetic joint infection using β-CD-conjugated indocyanine green in mice [48]. All studies with CD-based PAI tracers were conducted in small animals. Since the PAI tracers required irradiation with infrared light to emit ultrasound, further studies should move to the larger animals in order to evaluate penetration depth of the excitation light and to estimate the performance of PAI contrast agents prior to clinical translation. Furthermore, the development of larger pallet of PAI contrast dedicated for specific diseases would be beneficial to facilitate the future translation into the clinic.

The vast majority of the radiolabeled CDs derivatives were used for PET and SPECT imaging of the drug delivery and biodistribution in vivo. To be suitable for PET, multiple CD-based tracers containing ^64^Cu,^68^Ga, and ^18^F were developed. For SPECT imaging purposes, CD macrocycles were radiolabeled with either ^99m^Tc or ^125^I. The further widening of the radiolabeled CD derivatives might become a useful tool for pharmaceutics and drug development.

The most recent CD-based NPs were studied as contrast agents for CT. To be able to produce a sufficient contrast, the CD-based NPs must contain atoms with a high atomic number. The heavy atoms absorb X-rays with higher efficiency, increasing the X-ray attenuation coefficient of the tissue in which the contrast agent is present [107,108,109]. With modern advances in X-ray detection allowing lower dose image acquisition [110,111,112,113], the utilization of the novel CD-based NPs containing heavy elements will be highly beneficial for accurate anatomical imaging purposes. In addition, the novel dual energy CT approach [114,115] will benefit even more from the implementation of the novel CD-based NPs. Implementation and further development of the demonstrated NPs with substantially higher X-ray attenuation compared to currently available iodine and barium contrasts could potentially allow a superior improvement of CNR of the dual energy CT image. CT contrast of CD-based NPs was initially observed from contrast agents developed for multimodal imaging [50,53]. Only during the last three years has the research in synthesis of dedicated CT CD-based contrast agents been conducted extensively. Currently, the vast majority of the CD-based CT contrast agents were developed specifically for cancer imaging, whereas only one agent for angiography was developed. Therefore, it might be potentially useful to develop CD-based contrast agents suitable for different types of clinical CT imaging. In addition, the potential CD-based CT contrast agents must undergo an accurate toxicity study prior to further translation to in vivo imaging. Furthermore, special attention should be given to the investigation of extraction pathways of the contrast agents from the living organism during in vivo studies.

One of the potential approaches for further advances in the field of CD-based contrast agents is the development of a contrast agent that can serve for dual imaging modalities such as PET/MRI and PET/CT. Usually, the PET-active component of the tracer is small, and therefore, the development of the PET/MRI and PET/CT contrast agents could be built around radiolabeling of the existing CD-based agents for MRI and CT imaging modalities, respectively.

Overall, CDs are of high interest in the medical imaging field and are currently a very promising basis for developing various contrast agents. The successful clinical translation of CD-based contrast agents for proton MRI can help significantly improve the quality of clinical MRI scans. Further development of functionalized CD-based imaging agents for MRI has the potential to make molecular imaging using clinical proton MRI possible. Despite the enormous development level of CD-based contrast agents for conventional proton MRI, other imaging modalities started utilizing CD-based contrast agents recently and further developments and investigations are needed prior to successful clinical translation. Nevertheless, CD-based contrast agents demonstrated exceptional performance in the areas of CT, PET, and PAI.

## References

[1] Stella V.J., He Q. (2008). Cyclodextrins. Toxicol. Pathol..

[2] Jansook P., Ogawa N., Loftsson T. (2017). Cyclodextrins: Structure, physicochemical properties and pharmaceutical applications. Int. J. Pharm..

[3] Del Valle E.M.M. (2004). Cyclodextrins and their uses: A review. Process. Biochem..

[4] Lai W.-F. (2014). Cyclodextrins in non-viral gene delivery. Biomaterials.

[5] Irie T., Uekama K. (1997). Pharmaceutical Applications of Cyclodextrins. III. Toxicological Issues and Safety Evaluation. J. Pharm. Sci..

[6] Bellringer M., Smith T., Read R., Gopinath C., Olivier P. (1995). β-Cyclodextrin: 52-Week toxicity studies in the rat and dog. Food Chem. Toxicol..

[7] Loftsson T., Hreinsdóttir D., Másson M. (2005). Evaluation of cyclodextrin solubilization of drugs. Int. J. Pharm..

[8] Gidwani B., Vyas A. (2015). A Comprehensive Review on Cyclodextrin-Based Carriers for Delivery of Chemotherapeutic Cytotoxic Anticancer Drugs. BioMed Res. Int..

[9] Saokham P., Muankaew C., Jansook P., Loftsson T. (2018). Solubility of Cyclodextrins and Drug/Cyclodextrin Complexes. Molecules.

[10] Schönbeck C., Gaardahl K., Houston B. (2019). Drug Solubilization by Mixtures of Cyclodextrins: Additive and Synergistic Effects. Mol. Pharm..

[11] Jambhekar S.S., Breen P. (2016). Cyclodextrins in pharmaceutical formulations II: Solubilization, binding constant, and complexation efficiency. Drug Discov. Today.

[12] Loftsson T. (2017). Drug solubilization by complexation. Int. J. Pharm..

[13] Adeoye O., Cabral-Marques H. (2017). Cyclodextrin nanosystems in oral drug delivery: A mini review. Int. J. Pharm..

[14] Muankaew C., Loftsson T. (2018). Cyclodextrin-Based Formulations: A Non-Invasive Platform for Targeted Drug Delivery. Basic Clin. Pharmacol. Toxicol..

[15] Patel M.R., Lamprou D.A., Vavia P. (2020). Synthesis, Characterization, and Drug Delivery Application of Self-assembling Amphiphilic Cyclodextrin. AAPS PharmSciTech.

[16] Doan V.T.H., Lee J.H., Takahashi R., Nguyen P.T.M., Nguyen V.A.T., Pham H.T.T., Fujii S., Sakurai K. (2020). Cyclodextrin-based nanoparticles encapsulating α-mangostin and their drug release behavior: Potential carriers of α-mangostin for cancer therapy. Polym. J..

[17] Crini G., Fourmentin S., Fenyvesi É., Torri G., Fourmentin M., Morin-Crini N. (2018). Cyclodextrins, from molecules to applications. Environ. Chem. Lett..

[18] Lai W.-F. (2019). Design of cyclodextrin-based systems for intervention execution. Delivery of Therapeutics for Biogerontological Interventions.

[19] Davis M.E. (2009). The First Targeted Delivery of siRNA in Humans via a Self-Assembling, Cyclodextrin Polymer-Based Nanoparticle: From Concept to Clinic. Mol. Pharm..

[20] Malhotra M., Gooding M., Evans J.C., O’Driscoll D., Darcy R., O’Driscoll C.M. (2018). Cyclodextrin-siRNA conjugates as versatile gene silencing agents. Eur. J. Pharm. Sci..

[21] Wankar J., Kotla N.G., Gera S., Rasala S., Pandit A., Rochev Y.A. (2020). Recent Advances in Host–Guest Self-Assembled Cyclodextrin Carriers: Implications for Responsive Drug Delivery and Biomedical Engineering. Adv. Funct. Mater..

[22] Van De Manakker F., Vermonden T., Van Nostrum C.F., Hennink W.E. (2009). Cyclodextrin-Based Polymeric Materials: Synthesis, Properties, and Pharmaceutical/Biomedical Applications. Biomacromolecules.

[23] Feng Y., Xiao Q., Zhang Y., Li F., Li Y., Li C., Wang Q., Shi L., Lin H. (2017). Neodymium-doped NaHoF4 nanoparticles as near-infrared luminescent/T2-weighted MR dual-modal imaging agents in vivo. J. Mater. Chem. B.

[24] Ye M., Qian Y., Shen Y., Hu H., Sui M., Tang J. (2012). Facile synthesis and in vivo evaluation of biodegradable dendritic MRI contrast agents. J. Mater. Chem..

[25] Bartlett D.W., Su H., Hildebrandt I.J., Weber W.A., Davis M.E. (2007). Impact of tumor-specific targeting on the biodistribution and efficacy of siRNA nanoparticles measured by multimodality in vivo imaging. Proc. Natl. Acad. Sci. USA.

[26] Wang X., Parvathaneni V., Shukla S.K., Kanabar D.D., Muth A., Gupta V. (2020). Cyclodextrin Complexation for Enhanced Stability and Non-invasive Pulmonary Delivery of Resveratrol—Applications in Non-small Cell Lung Cancer Treatment. AAPS PharmSciTech.

[27] Rekharsky M.V., Inoue Y. (1998). Complexation Thermodynamics of Cyclodextrins. Chem. Rev..

[28] Hădărugă N.G., Bandur G.N., David I., Hădărugă D.I. (2018). A review on thermal analyses of cyclodextrins and cyclodextrin complexes. Environ. Chem. Lett..

[29] Liu L., Guo Q.-X. (2002). The Driving Forces in the Inclusion Complexation of Cyclodextrins. J. Incl. Phenom. Macrocycl. Chem..

[30] Hashidzume A., Yamaguchi H., Harada A. (2019). Cyclodextrin-Based Rotaxanes: From Rotaxanes to Polyrotaxanes and Further to Functional Materials. Eur. J. Org. Chem..

[31] Hashidzume A., Yamaguchi H., Harada A. (2014). Cyclodextrin-based molecular machines. Topics in Current Chemistry.

[32] Lai W.-F., Rogach A.L., Wong W.-Y. (2017). Chemistry and engineering of cyclodextrins for molecular imaging. Chem. Soc. Rev..

[33] Aime S., Botta M., Panero M., Grandi M., Uggeri F. (1991). Inclusion complexes between β-cyclodextrin and β-benzyloxy-α-propionic derivatives of paramagnetic DOTA- and DPTA-Gd(III) complexes. Magn. Reson. Chem..

[34] Aime S., Benetollo F., Bombieri G., Colla S., Fasano M., Paoletti S. (1997). Non-ionic Ln(III) chelates as MRI contrast agents: Synthesis, characterisation and 1H NMR relaxometric investigations of bis(benzylamide)diethylenetriaminepentaacetic acid Lu(III) and Gd(III) complexes. Inorg. Chim. Acta.

[35] Bryson J.M., Chu W.-J., Lee J.-H., Reineke T.M. (2008). A β-Cyclodextrin “Click Cluster” Decorated with Seven Paramagnetic Chelates Containing Two Water Exchange Sites. Bioconj. Chem..

[36] Barge A., Cravotto G., Robaldo B., Gianolio E., Aime S. (2007). New CD derivatives as self-assembling contrast agents for magnetic resonance imaging (MRI). J. Incl. Phenom. Macrocycl. Chem..

[37] Carrera C., Digilio G., Baroni S., Burgio D., Consol S., Fedeli F., Longo D.L., Mortillaro A., Aime S. (2007). Synthesis and characterization of a Gd(iii) based contrast agent responsive to thiol containing compounds. Dalton Trans..

[38] Gomes P.M.O., Silva A.M., Silva V.L. (2020). Pyrazoles as Key Scaffolds for the Development of Fluorine-18-Labeled Radiotracers for Positron Emission Tomography (PET). Molecules.

[39] Liu Q., Chen M., Sun Y., Chen G., Yang T., Gao Y., Zhang X., Li F. (2011). Multifunctional rare-earth self-assembled nanosystem for tri-modal upconversion luminescence /fluorescence /positron emission tomography imaging. Biomaterials.

[40] Hajdu I., Angyal J., Szikra D., Kertész I., Malanga M., Fenyvesi É., Szente L., Vecsernyés M., Bácskay I., Váradi J. (2019). Radiochemical synthesis and preclinical evaluation of 68Ga-labeled NODAGA-hydroxypropyl-beta-cyclodextrin (68Ga-NODAGA-HPBCD). Eur. J. Pharm. Sci..

[41] Trencsényi G., Kis A., Szabó J.P., Ráti Á., Csige K., Fenyvesi É., Szente L., Malanga M., Méhes G., Emri M. (2020). In vivo preclinical evaluation of the new 68Ga-labeled beta-cyclodextrin in prostaglandin E2 (PGE2) positive tumor model using positron emission tomography. Int. J. Pharm..

[42] Areses P., Agüeros M.T., Quincoces G., Collantes M., Richter J.Á., López-Sánchez L.M., Sánchez-Martínez M., Irache J.M., Peñuelas I. (2011). Molecular Imaging Techniques to Study the Biodistribution of Orally Administered 99mTc-Labelled Naive and Ligand-Tagged Nanoparticles. Mol. Imaging Biol..

[43] Perret P., Bacot S., Gèze A., Maurin A.G.D., Debiossat M., Soubies A., Blanc-Marquis V., Choisnard L., Boutonnat J., Ghezzi C. (2018). Biodistribution and preliminary toxicity studies of nanoparticles made of Biotransesterified β–cyclodextrins and PEGylated phospholipids. Mater. Sci. Eng. C.

[44] Yao Y., Liu X., Liu T., Zhou J., Zhu J., Sun G., He D. (2015). Preparation of inclusion complex of perfluorocarbon compound with β-cyclodextrin for ultrasound contrast agent. RSC Adv..

[45] Weber J., Beard P.C., Bohndiek S.E. (2016). Contrast agents for molecular photoacoustic imaging. Nat. Methods.

[46] Laramie M.D., Smith M.K., Marmarchi F., McNally L.R., Henary M. (2018). Small Molecule Optoacoustic Contrast Agents: An Unexplored Avenue for Enhancing In Vivo Imaging. Molecules.

[47] Yu Z., Wang M., Pan W., Wang H., Li N., Tang B. (2017). Tumor microenvironment-triggered fabrication of gold nanomachines for tumor-specific photoacoustic imaging and photothermal therapy. Chem. Sci..

[48] Wang Y., Thompson J.M., Ashbaugh A.G., Khodakivskyi P., Budin G., Sinisi R., Heinmiller A., Van Oosten M., Van Dijl J.M., Van Dam G.M. (2017). Preclinical Evaluation of Photoacoustic Imaging as a Novel Noninvasive Approach to Detect an Orthopaedic Implant Infection. J. Am. Acad. Orthop. Surg..

[49] Maji S.K., Sreejith S., Joseph J., Lin M., He T., Tong Y., Sun H., Yu S.W.-K., Zhao Y. (2014). Upconversion Nanoparticles as a Contrast Agent for Photoacoustic Imaging in Live Mice. Adv. Mater..

[50] Yin W., Tian G., Ren W., Yan L., Jin S., Gu Z., Zhou L., Li J., Zhao Y. (2014). Design of multifunctional alkali ion doped CaF2 upconversion nanoparticles for simultaneous bioimaging and therapy. Dalton Trans..

[51] Lin W., Zhang X., Qian L., Yao N., Pan Y., Zhang L. (2017). Doxorubicin-Loaded Unimolecular Micelle-Stabilized Gold Nanoparticles as a Theranostic Nanoplatform for Tumor-Targeted Chemotherapy and Computed Tomography Imaging. Biomacromolecules.

[52] Zhang C., Wang S.-B., Chen Z.-X., Fan J.-X., Zhong Z., Zhang X.-Z. (2019). A tungsten nitride-based degradable nanoplatform for dual-modal image-guided combinatorial chemo-photothermal therapy of tumors. Nanoscale.

[53] Zhou J., Lu Z., Shan G., Wang S., Liao Y. (2014). Gadolinium complex and phosphorescent probe-modified NaDyF4 nanorods for T1- and T2-weighted MRI/CT/phosphorescence multimodality imaging. Biomaterials.

[54] Champagne P.-L., Barbot C., Zhang P., Han X., Gaamoussi I., Hubert-Roux M., Bertolesi G.E., Gouhier G., Ling C.-C. (2018). Synthesis and Unprecedented Complexation Properties of β-Cyclodextrin-Based Ligand for Lanthanide Ions. Inorg. Chem..

[55] Kotková Z., Kotek J., Jirák D., Jendelová P., Herynek V., Berková Z., Hermann P., Lukeš I. (2010). Cyclodextrin-Based Bimodal Fluorescence/MRI Contrast Agents: An Efficient Approach to Cellular Imaging. Chem. A Eur. J..

[56] Kotková Z., Helm L., Kotek J., Hermann P., Lukeš I. (2012). Gadolinium complexes of monophosphinic acid DOTA derivatives conjugated to cyclodextrin scaffolds: Efficient MRI contrast agents for higher magnetic fields. Dalton Trans..

[57] Freed J.H. (1978). Dynamic effects of pair correlation functions on spin relaxation by translational diffusion in liquids. II. Finite jumps and independent T1 processes. J. Chem. Phys..

[58] Aime S., Botta M., Frullano L., Crich S.G., Giovenzana G.B., Pagliarin R., Palmisano G., Sisti M. (1999). Contrast Agents for Magnetic Resonance Imaging: A Novel Route to Enhanced Relaxivities Based on the Interaction of a Gd III Chelate with Poly-b-cyclodextrins. Chem. Eur. J..

[59] Aime S., Botta M., Fedeli F., Gianolio E., Terreno E., Anelli P. (2001). High-Relaxivity Contrast Agents for Magnetic Resonance Imaging Based on Multisite Interactions between a β-Cyclodextrin Oligomer and Suitably Functionalized Gd III Chelates. Chem. Eur. J..

[60] Battistini E., Gianolio E., Gref R., Couvreur P., Füzerová S., Othman M., Aime S., Badet B., Durand P., Patrick C. (2008). High-Relaxivity Magnetic Resonance Imaging (MRI) Contrast Agent Based on Supramolecular Assembly between a Gadolinium Chelate, a Modified Dextran, and Poly-β-Cyclodextrin. Chem. A Eur. J..

[61] Martinelli J., Fekete M., Tei L., Botta M. (2011). Cleavable β-cyclodextrin nanocapsules incorporating GdIII-chelates as bioresponsive MRI probes. Chem. Commun..

[62] Lahrech H., Perles-Barbacaru A.-T., Aous S., Le Bas J.-F., Debouzy J.-C., Gadelle A., Fries P.H. (2008). Cerebral Blood Volume Quantification in a C6 Tumor Model Using Gadolinium per (3,6-Anhydro) α-Cyclodextrin as a New Magnetic Resonance Imaging Preclinical Contrast Agent. J. Cereb. Blood Flow Metab..

[63] Sun M., Zhang H.-Y., Liu B.-W., Liu Y. (2013). Construction of a Supramolecular Polymer by Bridged Bis(permethyl-β-cyclodextrin)s with Porphyrins and Its Highly Efficient Magnetic Resonance Imaging. Macromolecules.

[64] Sun M., Zhang H., Hu X., Liu B., Liu Y. (2014). Hyperbranched Supramolecular Polymer of Tris(permethyl-β-cyclodextrin)s with Porphyrins: Characterization and Magnetic Resonance Imaging. Chin. J. Chem..

[65] Gomori J.M., I Grossman R., Yu-Ip C., Asakura T. (1987). NMR relaxation times of blood: Dependence on field strength, oxidation state, and cell integrity. J. Comput. Assist. Tomogr..

[66] Peters A.M., Brookes M.J., Hoogenraad F.G., Gowland P.A., Francis S.T., Morris P.G., Bowtell R. (2007). T2* measurements in human brain at 1.5, 3 and 7 T. Magn. Reson. Imaging.

[67] Zhou Z., Han Z., Lu Z.-R. (2016). A targeted nanoglobular contrast agent from host–guest self-assembly for MR cancer molecular imaging. Biomaterials.

[68] Liu T., Li X., Qian Y., Hu X., Liu S. (2012). Multifunctional pH-Disintegrable micellar nanoparticles of asymmetrically functionalized β-cyclodextrin-Based star copolymer covalently conjugated with doxorubicin and DOTA-Gd moieties. Biomaterials.

[69] Skinner P.J., Beeby A., Dickins R.S., Parker D., Aime S., Botta M. (2000). Conjugates of cyclodextrins with charged and neutral macrocyclic europium, terbium and gadolinium complexes: Sensitised luminescence and relaxometric investigations and an example of supramolecular relaxivity enhancement. J. Chem. Soc. Perkin Trans. 2.

[70] Fredy J.W., Scelle J., Guenet A., Morel E., De Beaumais S.A., Ménand M., Marvaud V., Bonnet C.S., Tóth É., Sollogoub M. (2014). Cyclodextrin Polyrotaxanes as a Highly Modular Platform for the Development of Imaging Agents. Chem. A Eur. J..

[71] Su H., Liu Y., Wang D., Wu C., Xia C., Gong Q., Song B., Ai H. (2013). Amphiphilic starlike dextran wrapped superparamagnetic iron oxide nanoparticle clsuters as effective magnetic resonance imaging probes. Biomaterials.

[72] Oroujeni M., Kaboudin B., Xia W., Jönsson P., Ossipov D.A. (2018). Conjugation of cyclodextrin to magnetic Fe3O4 nanoparticles via polydopamine coating for drug delivery. Prog. Org. Coat..

[73] Zhou Z., Mondjinou Y., Hyun S.-H., Kulkarni A., Lu Z.-R., Thompson D.H. (2015). Gd3+-1,4,7,10-Tetraazacyclododecane-1,4,7-triacetic-2-hydroxypropyl-β-cyclodextrin/Pluronic Polyrotaxane as a Long Circulating High Relaxivity MRI Contrast Agent. ACS Appl. Mater. Interfaces.

[74] Zhou X., Ye M., Han Y., Tang J., Qian Y., Hu H., Shen Y. (2017). Enhancing MRI of liver metastases with a zwitterionized biodegradable dendritic contrast agent. Biomater. Sci..

[75] Zhang Q., Lu Y., Xu X., Li S., Du Y., Yu R.-S. (2019). MR molecular imaging of HCC employing a regulated ferritin gene carried by a modified polycation vector. Int. J. Nanomed..

[76] Mortezazadeh T., Gholibegloo E., Alam N.R., Dehghani S., Haghgoo S., Ghanaati H., Khoobi M. (2019). Gadolinium (III) oxide nanoparticles coated with folic acid-functionalized poly(β-cyclodextrin-co-pentetic acid) as a biocompatible targeted nano-contrast agent for cancer diagnostic: In vitro and in vivo studies. MAGMA Magn. Reson. Mater. Phys. Biol. Med..

[77] Han Y., Zhou X., Qian Y., Hu H., Zhou Z., Liu X., Tang J., Shen Y. (2019). Hypoxia-targeting dendritic MRI contrast agent based on internally hydroxy dendrimer for tumor imaging. Biomaterials.

[78] Hane F.T., Robinson M., Lee B.Y., Bai O., Leonenko Z., Albert M.S. (2017). Recent Progress in Alzheimer’s Disease Research, Part 3: Diagnosis and Treatment. J. Alzheimers Dis..

[79] Schröder L., Lowery T.J., Hilty C., Wemmer D.E., Pines A. (2006). Molecular Imaging Using a Targeted Magnetic Resonance Hyperpolarized Biosensor. Science.

[80] Hane F.T., Li T., Smylie P., Pellizzari R.M., Plata J.A., DeBoef B., Albert M.S. (2017). In vivo detection of cucurbit[6]uril, a hyperpolarized xenon contrast agent for a xenon magnetic resonance imaging biosensor. Sci. Rep..

[81] Albert M.S., Cates G.D., Driehuys B., Happer W., Saam B., Springer C.S., Wishnia A. (1994). Biological magnetic resonance imaging using laser-polarized 129Xe. Nat. Cell Biol..

[82] Norquay G., Collier G.J., Rao M., Stewart N.J., Wild J.M. (2018). Xe129 -Rb Spin-Exchange Optical Pumping with High Photon Efficiency. Phys. Rev. Lett..

[83] Nikolaou P., Coffey A.M., Walkup L.L., Gust B.M., Whiting N., Newton H., Barcus S., Muradyan I., Dabaghyan M., Moroz G.D. (2013). Near-unity nuclear polarization with an open-source 129Xe hyperpolarizer for NMR and MRI. Proc. Natl. Acad. Sci. USA.

[84] Comment A., Jannin S., Hyacinthe J.-N., Miéville P., Sarkar R., Ahuja P., Vasos P.R., Montet X., Lazeyras F., Vallee J.-P. (2010). Hyperpolarizing Gases via Dynamic Nuclear Polarization and Sublimation. Phys. Rev. Lett..

[85] Kurhanewicz J., Vigneron D.B., Ardenkjaer-Larsen J.H., Bankson J.A., Brindle K., Cunningham C.H., Gallagher F.A., Keshari K.R., Kjaer A., Laustsen C. (2019). Hyperpolarized 13C MRI: Path to Clinical Translation in Oncology. Neoplasia.

[86] Keshari K.R., Kurhanewicz J., Macdonald J.M., Wilson D.M. (2012). Generating contrast in hyperpolarized 13C MRI using ligand–receptor interactions. Analyst.

[87] Caracciolo F., Carretta P., Filibian M., Melone L. (2017). Dynamic Nuclear Polarization of β-Cyclodextrin Macromolecules. J. Phys. Chem. B.

[88] Caracciolo F., Paioni A.L., Filibian M., Melone L., Carretta P. (2018). Proton and Carbon-13 Dynamic Nuclear Polarization of Methylated β-Cyclodextrins. J. Phys. Chem. B.

[89] Wang Y., Dmochowski I.J. (2016). An Expanded Palette of Xenon-129 NMR Biosensors. Acc. Chem. Res..

[90] Shapiro M.G., Ramirez R.M., Sperling L.J., Sun G., Sun J., Pines A., Schaffer D.V., Bajaj V.S. (2014). Genetically encoded reporters for hyperpolarized xenon magnetic resonance imaging. Nat. Chem..

[91] Song Y.-Q., Goodson A.P.B.M., Taylor R.E., Laws D.D., Navon G., Pines A. (1997). Selective Enhancement of NMR Signals forα-Cyclodextrin with Laser-Polarized Xenon. Angew. Chem. Int. Ed..

[92] Karas S. (2016). The Synthesis of Rotaxane Probes for Magnetic Resonance Imaging (MRI). Master’s Thesis.

[93] Hane F.T., Smylie P.S., Julia R., Ruberto J., Dowhos K., Ball I., Tomanek B., DeBoef B., Albert M.S. (2016). HyperCEST detection of cucurbit[6]uril in whole blood using an ultrashort saturation Pre-pulse train. Contrast Media Mol. Imaging.

[94] Finbloom J.A., Slack C.C., Bruns C.J., Jeong K., Wemmer D.E., Pines A., Francis M.B. (2016). Rotaxane-mediated suppression and activation of cucurbit[6]uril for molecular detection by 129Xe hyperCEST NMR. Chem. Commun..

[95] Hane F.T., Fernando A., Prete B.R.J., Peloquin B., Karas S., Chaudhuri S., Chahal S., Shepelytskyi Y., Wade A., Li T. (2018). Cyclodextrin-Based Pseudorotaxanes: Easily Conjugatable Scaffolds for Synthesizing Hyperpolarized Xenon-129 Magnetic Resonance Imaging Agents. ACS Omega.

[96] Cavalieri F., El Hamassi A., Chiessi E., Paradossi G., Villa R., Zaffaroni N. (2006). Tethering Functional Ligands onto Shell of Ultrasound Active Polymeric Microbubbles. Biomacromolecules.

[97] Yeo S.K., Shepelytskyi Y., Grynko V., Albert M.S. (2020). Molecular Imaging of Fluorinated Probes for Tau Protein and Amyloid-β Detection. Molecules.

[98] Schluep T., Hwang J., Hildebrandt I.J., Czernin J., Hang C., Choi J., Alabi C.A., Mack B.C., Davis M.E. (2009). Pharmacokinetics and tumor dynamics of the nanoparticle IT-101 from PET imaging and tumor histological measurements. Proc. Natl. Acad. Sci. USA.

[99] Hou S., Choi J.-S., Garcia M.A., Xing Y., Chen K.-J., Chen Y.-M., Jiang Z.K., Ro T., Wu L., Stout D.B. (2016). Pretargeted Positron Emission Tomography Imaging That Employs Supramolecular Nanoparticles with in Vivo Bioorthogonal Chemistry. ACS Nano.

[100] Yaméogo J.B.G., Géze A., Choisnard L., Putaux J.-L., Semdé R., Wouessidjewe D. (2014). Progress in developing amphiphilic cyclodextrin-based nanodevices for drug delivery. Curr. Top. Med. Chem..

[101] Yaméogo J.B., Gèze A., Choisnard L., Putaux J.-L., Mazet R., Passirani C., Keramidas M., Coll J.-L., Lautram N., Bejaud J. (2014). Self-assembled biotransesterified cyclodextrins as potential Artemisinin nanocarriers. II: In vitro behavior toward the immune system and in vivo biodistribution assessment of unloaded nanoparticles. Eur. J. Pharm. Biopharm..

[102] Yu G., Yang Z., Fu X., Yung B.C., Yang J., Mao Z., Shao L., Hua B., Liu Y., Zhang F. (2018). Polyrotaxane-based supramolecular theranostics. Nat. Commun..

[103] Sauer R.-S., Rittner H.L., Roewer N., Sohajda T., Shityakov S., Brack A., Broscheit J.-A. (2017). A Novel Approach for the Control of Inflammatory Pain: Prostaglandin E2 Complexation by Randomly Methylated β-Cyclodextrins. Anesth. Analg..

[104] Zhu C., Ma X., Ma D., Zhang T., Gu N. (2018). Crosslinked Dextran Gel Microspheres with Computed Tomography Angiography and Drug Release Function. J. Nanosci. Nanotechnol..

[105] Lin W., Yao N., Qian L., Zhang X., Chen Q., Wang J., Zhang L.-J. (2017). pH-responsive unimolecular micelle-gold nanoparticles-drug nanohybrid system for cancer theranostics. Acta Biomater..

[106] Lin W., Yang C., Xue Z., Huang Y., Luo H., Zu X., Zhang L., Yi G. (2018). Controlled construction of gold nanoparticles in situ from β-cyclodextrin based unimolecular micelles for in vitro computed tomography imaging. J. Colloid Interface Sci..

[107] Lee N., Choi S.H., Hyeon T. (2013). Nano-Sized CT Contrast Agents. Adv. Mater..

[108] Xi D., Dong S., Meng X., Lu Q., Meng L., Ye J. (2012). Gold nanoparticles as computerized tomography (CT) contrast agents. RSC Adv..

[109] Shilo M., Reuveni T., Motiei M., Popovtzer R. (2012). Nanoparticles as computed tomography contrast agents: Current status and future perspectives. Nanomedicine.

[110] Gu J., Bednarz B., Caracappa P.F., Xu X.G. (2009). The development, validation and application of a multi-detector CT (MDCT) scanner model for assessing organ doses to the pregnant patient and the fetus using Monte Carlo simulations. Phys. Med. Biol..

[111] Semeniuk O., Grynko O., DeCrescenzo G., Juska G., Wang K., Reznik A. (2017). Characterization of polycrystalline lead oxide for application in direct conversion X-ray detectors. Sci. Rep..

[112] Wolterink J.M., Leiner T., Viergever M.A., Isgum I. (2017). Generative Adversarial Networks for Noise Reduction in Low-Dose CT. IEEE Trans. Med. Imaging.

[113] Grynko O., Juska G., Reznik A. An Engineering of multilayered lead oxide photoconductor for lag-free X-ray digital detector. Proceedings of the IEEE Nuclear Science Symposium and Medical Imaging Conference (NSS/MIC).

[114] Patino M., Prochowski A., Agrawal M.D., Simeone F.J., Gupta R., Hahn P.F., Sahani D.V. (2016). Material Separation Using Dual-Energy CT: Current and Emerging Applications. Radiographics.

[115] Albrecht M.H., Vogl T.J., Martin S.S., Nance J.W., Duguay T.M., Wichmann J.L., De Cecco C.N., Varga-Szemes A., Van Assen M., Tesche C. (2019). Review of Clinical Applications for Virtual Monoenergetic Dual-Energy CT. Radiology.

